# Neutrophil infiltration and whole-cell vaccine elicited by N-dihydrogalactochitosan combined with NIR phototherapy to enhance antitumor immune response and T cell immune memory

**DOI:** 10.7150/thno.38515

**Published:** 2020-01-01

**Authors:** Shuhong Qi, Lisen Lu, Feifan Zhou, Yuzhou Chen, Mengli Xu, Lu Chen, Xiang Yu, Wei R. Chen, Zhihong Zhang

**Affiliations:** 1 Britton Chance Center for Biomedical Photonics, Wuhan National Laboratory for Optoelectronics-Huazhong University of Science and Technology, Wuhan, Hubei 430074, China; 2 MoE Key Laboratory for Biomedical Photonics, Collaborative Innovation Center for Biomedical Engineering, School of Engineering Sciences, Huazhong University of Science and Technology, Wuhan, Hubei 430074, China; 3 Biophotonics Research Laboratory, Center for Interdisciplinary Biomedical Education and Research, College of Mathematics and Science, University of Central Oklahoma, Edmond, Oklahoma 73034, United States

**Keywords:** laser immunotherapy, intravital imaging, whole-cell vaccine, immune memory

## Abstract

Melanoma is one of the deadliest malignancies with a high risk of relapse and metastasis. Long-term, tumor-specific, and systemic immunity induced by local intervention is ideal for personalized cancer therapy. Laser immunotherapy (LIT), a combination of local irradiation of laser and local administration of an immunostimulant, was developed to achieve such an immunity. Although LIT showed promising efficacy on tumors, its immunological mechanism is still not understood, especially its spatio-temporal dynamics.

**Methods**: In this study, we investigated LIT-induced immunological responses using a 980-nm laser and a novel immunostimulant, N-dihydrogalactochitosan (GC). Then we followed the functions of key immune cells spatially and temporally using intravital imaging and immunological assays.

**Results**: Immediately after LIT, GC induced a rapid infiltration of neutrophils which ingested most GC in tumors. The cytokines released to the serum peaked at 12 h after LIT. Laser irradiations produced photothermal effects to ablate the tumor, release damage-associated molecular patterns, and generate whole-cell tumor vaccines. LIT-treated tumor-bearing mice efficiently resisted the rechallenged tumor and prevented lung metastasis. Intravital imaging of tumor at rechallenging sites in LIT-treated mice revealed that the infiltration of tumor-infiltrating lymphocytes (TILs) increased with highly active motility. Half of TILs with arrest and confined movements indicated that they had long-time interactions with tumor cells. Furthermore, LIT has synergistic effect with checkpoint blockade to improve antitumor efficacy.

**Conclusion**: Our research revealed the important role of LIT-induced neutrophil infiltration on the *in situ* whole-cell vaccine-elicited antitumor immune response and long-term T cell immune memory.

## Introduction

Melanoma is one of the deadliest and treatment-resistant malignancy, and its incidence has been rising in recent years 1. Although surgery is considered as the standard therapy, it increases the risk of metastasis and recurrence 2, 3. Once melanoma relapses and/or metastasizes, it is extremely hard to treat, resulting in high mortality 4. Therefore, one of the biggest challenges facing melanoma treatment is the control of relapse and metastasis. Recently, immunotherapy has made significant progress and is considered as the new hope of cancer patients 5, 6. Current promising immunotherapies include cytokine and monoclonal antibody therapy, adoptive cell therapy (ACT), checkpoint blockade therapy, and tumor vaccines 5, 6. Up to now, these immunotherapies still have some limitations, such as high cost, immune toxicity like “cytokine storm” 7-9, and over-activation of the immune system (acute diabetes and myocarditis 10, 11).

Among cancer immunotherapies, personalized cancer vaccines have received extensive attention in recent years 12, 13. Especially, whole-cell cancer vaccine has been accepted by most researchers due to the following advantages: (1) the vaccine is loaded with each patient's all tumor antigens to induce a specific antitumor immune response to achieve personalized immunotherapy 12, 14; (2) the *ex vivo* screening of tumor-specific antigens is not needed because the tumor cells contain all potential antigens 14; (3) the long-term immune memory produced by whole-cell cancer vaccines can prevent tumor recurrence effectively and inhibit tumor metastasis 13. However, the drawback for cancer vaccines is that they have the potential to induce high expression of programmed death ligand 1 (PD-L1) on tumor cells, which enables these cells to escape the attack by immune cells 15 .

Photothermal therapy (PTT) is a unique cancer therapeutic strategy, that converts absorbed light energy into heat to ablate solid tumors 16-18. Local PTT treatment induces immunogenic tumor cell death by producing damage-associated molecular patterns (DAMPs) to further elicit antitumor immune responses. The advantages of PTT include being easy-to-operate, safe, and having low toxicity and limited side-effects. Nevertheless, laser radiation induced photothermal effects and immune responses are not strong enough to eliminate the tumors and prevent the relapse and metastasis. Thus, additional sensitizers and immunostimulants are needed, especially nanoparticles that can improve the distribution of sensitizers and immunostimulants in tumors to achieve enhanced antitumor immune responses 19, 20.

N-dihydrogalactochitosan (GC) is a nontoxic, biocompatible and biodegradable polysaccharide that is used as a potential stimulant for vaccines. Laser immunotherapy (LIT), using laser irradiation, followed by intratumoral injection of GC, was developed to treat metastatic mammary tumors *in situ* 21, 22. GC has been shown to be able to stimulate immature DCs* in vitro* 23 and recruited T cells into the treated tumors *in vivo* when combined with laser irradiation 24. LIT has been administrated to treat various tumor models by using different cell lines, such as Panc02-H7 pancreatic tumor cells 24, EMT6 murine mammary tumor cells 25, and cutaneous squamous cell carcinoma A431 tumor cells 26. In addition, LIT has been used in preliminary clinical trials to treat melanoma and breast cancer patients 27-29. Particularly, when LIT was used in conjunction with a checkpoint inhibitor (anti-CTLA-4), it has been highly effective for late-stage, metastatic melanoma patients, eradicating treated surface melanoma lesions and untreated lung metastasis 29. Although previous preclinical and clinical experiments have proven that the LIT has a promising curative effect on tumors, its immunological mechanism and time-series change are still not clear, especially the spatio-temporal information of activated T cells on distant tumors. The immunomodulatory effect of GC reportedly includes modulating macrophage polarization, influencing dendritic cell activation, and stimulating adaptive T cells 30, 31. Although some immunological properties of GC have been exposed, the direct targets of GC *in vivo*, and how GC modulates immune response are not fully understood.

In this study, melanoma-bearing mice were treated with an intratumoral injection of GC, and local irradiation by a 980-nm laser 2 h later. We investigated antitumor effects induced by LIT (also labeled as GC + PTT in the following paragraphs), including its curative effect, cancer vaccine-like functionality, and long-term antitumor immune responses. Using immune assays and intravital imaging, we determined the timelines of antitumor immune responses in the treated primary tumors, tumor-draining lymph nodes (TDLNs), and rechallenged tumors after LIT. Interestingly, we found that the intratumoral injection of GC induced a rapid infiltration of neutrophils, and most of the GCs were ingested by neutrophils. Combined with PTT, a significant number of neutrophils was recruited into the treated tumors at the early stage after LIT, which accelerated the generation of the whole-cell vaccine to elicit systematic antitumor immune response. The combined application of the checkpoint-blockade with LIT decreased the PD-L1 on tumor cells, reversed the dysfunction of cytotoxic T cells (CTLs), and inhibited the distant secondary B16 tumors efficiently.

## Results

### Local PTT treatment caused B16 tumor regression and LIT enhanced the survival of tumor-bearing mice

**Figure 1:**
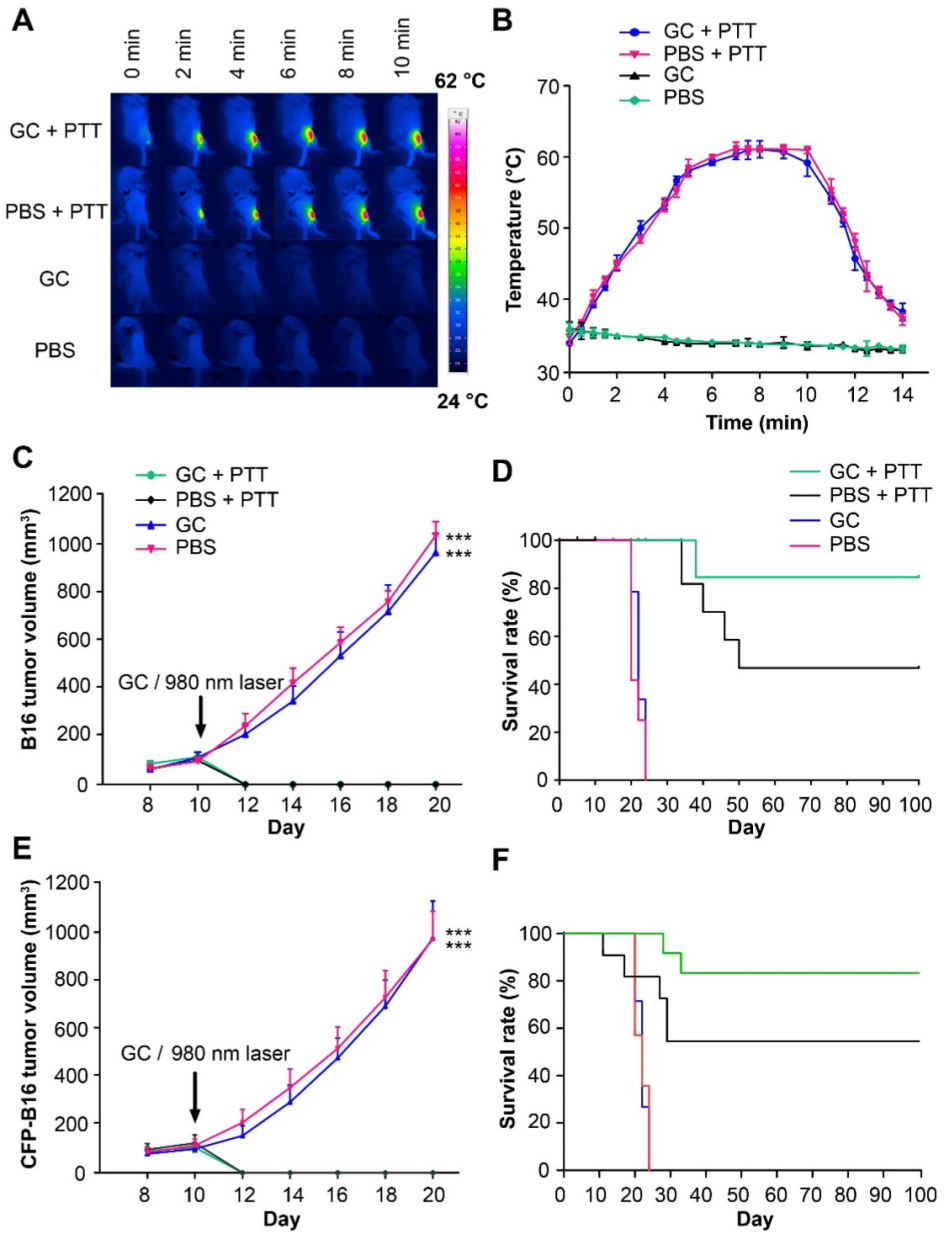
** The treatments of subcutaneous B16 and CFP-B16 tumors. (A)** Infrared (IR) thermal images of mice bearing CFP-B16 tumors under different treatments (GC + PTT, PBS + PTT, GC, or PBS). **(B)** Tumor temperature changes based on IR thermal imaging data in **(A)**. Data are presented as mean ± SD (n = 3 mice). **(C)** Volume of B16 tumors in the mice of different treatment groups. Data are presented as mean ± SD (n = 9-10 mice, two independent experiments, PBS *versus* GC + PTT, *** *P* < 0.001, and GC *versus* GC + PTT, *** *P* < 0.001). **(D)** Survival rates of mice bearing B16 tumors after various treatments (9-10 mice per group). **(E)** Volume of CFP-B16 tumors in the mice of different treatment groups. Data are presented as mean ± SD (n = 10 mice, two independent experiments, GC + PTT *versus* PBS, *** *P* < 0.001, and GC + PTT* versus* GC, *** *P* < 0.001). **(F)** Survival rates of mice bearing CFP-B16 tumors after various treatments (10 mice per group). Statistical analysis was performed using the Kruskal-Wallis test followed by Dunn's multiple comparison tests and the log-rank Mantel-Cox test.

We used B16 or CFP-B16 (B16 tumor cells stably expressing the mutant of cyan fluorescent protein mCerulean), both poorly immunogenic 32, to prepare the tumor model in C57BL/6 mice. B16 or CFP-B16 subcutaneous tumors were intratumorally injected with 100 μl 1% GC (the structure of GC is shown in Figure S1) or PBS, and 2 h later irradiated by a 980-nm laser (power density: 1 W/cm^2^) for 10 min. Monitoring by an infrared thermal camera revealed that the tumor temperature increased quickly and stabilized at 60 °C in both GC + PTT and PBS + PTT groups (Figure 1A-B). The primary B16 tumors in the GC + PTT and PBS + PTT groups regressed rapidly (2-3 days) after laser irradiation, as shown in Figure 1C, demonstrating the effectiveness of local PTT on primary tumors. Survival rates of mice were monitored for 100 days. In the GC + PTT group, 8 of 10 mice survived and remained tumor-free for 100 days. However, only 4 of 9 mice in the PBS + PTT group survived. In comparison, all the mice treated with PBS or GC-alone died within 24 days after treatment (Figure 1D).

For the subsequent intravital optical imaging of tumor microenvironment, we also monitored the growth of CFP-B16 tumors and the survival rates of mice under different treatments. Similar to B16 tumors, the CFP-B16 tumors in the GC + PTT and PBS + PTT treated mice regressed quickly (2-3 days) after treatment (Figure 1E). In the GC + PTT group, 8 of 10 mice survived and remained tumor free for 100 days, and in the PBS + PTT group, 6 of 10 mice survived. In contrast, all the mice treated with PBS or GC-alone died within 24 days after treatment (Figure 1F). These results demonstrated the effectiveness of LIT in treating primary melanoma *in situ*.

### GC-induced neutrophil infiltration enhanced the LIT-elicited antitumor immune response

**Figure 2:**
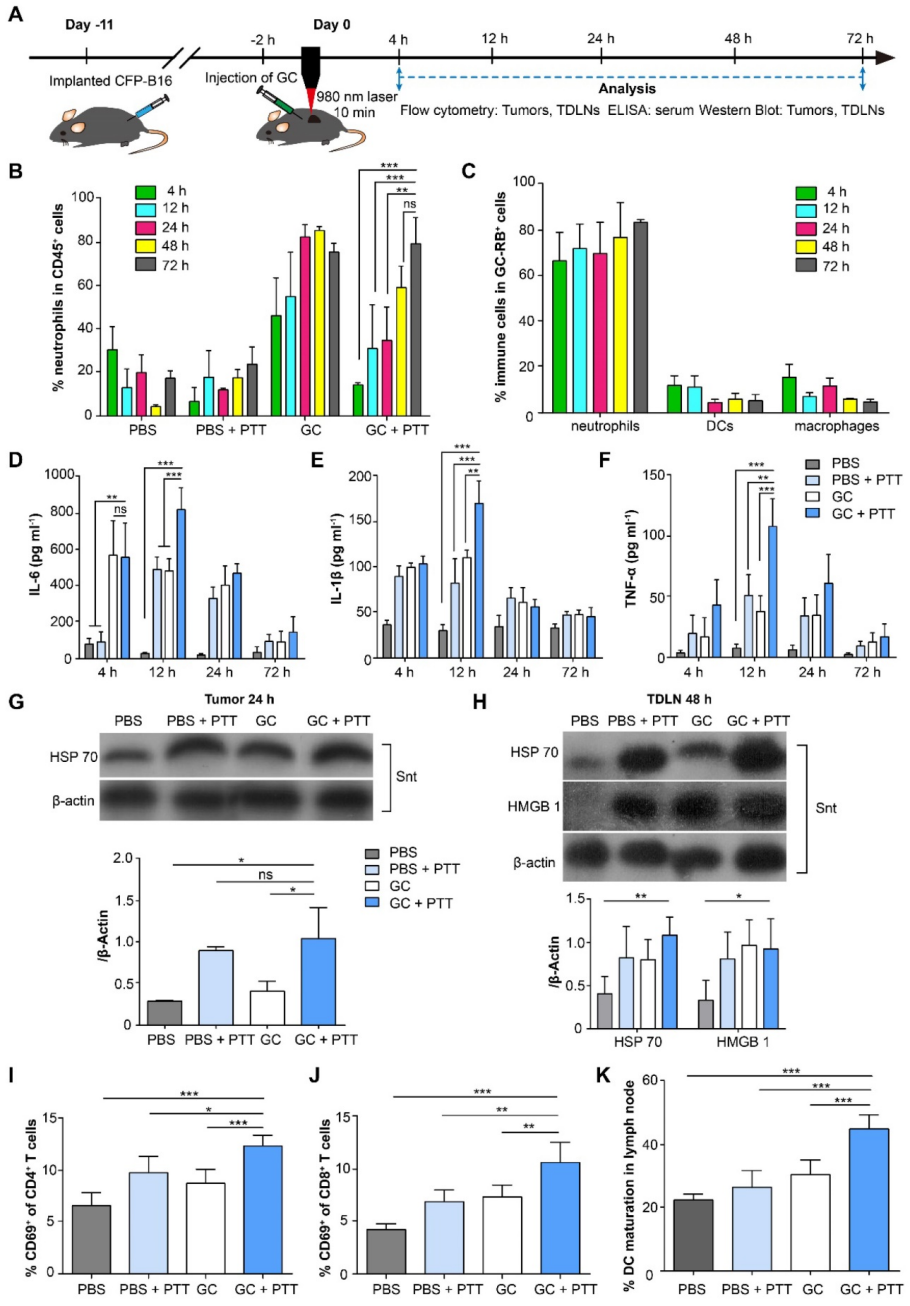
** Immunological responses induced by LIT in the tumors, serum, and TDLNs. (A)** Schematics of the procedures and timeline of GC + PTT treatment for CFP-B16 and analysis of LIT-induced antitumor immune response.** (B)** Proportions of neutrophils in immune cells in the treated primary tumors after various treatments at different times. **(C)** Proportions of neutrophils in immune cells with GC-RB (gated by CD45^+^ and RB^+^) in the tumors treated with GC-RB + PTT at different times. Data are presented as mean ± SD (n = 3-5 mice, two independent experiments).** (D-F)** Cytokine levels in serum (TNF-α, IL-6, and IL-1β) from mice at different times after various treatments. Data are presented as mean ± SD (n = 3-4 mice, two independent experiments). **(G)** HSP70 protein expression in the treated primary tumors 24 h after various treatments was analyzed using WB. **(H)** HSP70 and HMGB1 expressions in TDLNs at 48 h after different treatments were analyzed using WB (n = 3, two independent experiments). **(I, J)** The frequency of CD69^+^ in the CD4^+^ **(I)** and CD8^+^ **(J)** T cells of TDLNs 24 h after different treatments. **(K)** The frequency of mature DCs (CD11c^+^CD80^+^CD86^+^) in TDLNs 72 h after various treatments. Data are presented as mean ± SD (n = 4-7 mice, three independent experiments). Statistical analysis was performed using the unpaired *t*-test, and the one-way ANOVA test followed by the Bonferroni post-test. * *P* < 0.05, ** *P* < 0.01, *** *P* < 0.001, ns: not significant.

To investigate the immune response induced by LIT, we analyzed both innate and adaptive immunity in treated tumors, serum, and tumor-draining lymph nodes (TDLNs). The experimental procedures and timeline of immunological analyses are shown in Figure 2A. Firstly, we analyzed innate immune cells, including neutrophils, macrophages, and dendritic cells (DCs), in the tumors at different time points after treatments (Figure 2B and Figure S2). As shown in Figure 2B, the proportion of neutrophils continued to increase and remained high from the 24-h (83.4%) to the 72-h (76.4%) time points in the GC-alone treated tumors. Although laser irradiation promoted the proportion of neutrophils in the GC + PTT treated tumors to decrease significantly (compared with GC-alone group) at 4 h (14.5%, Figure 2B), the amount of neutrophils kept increasing and reached a high level at the 48-h (60%) and 72-h (80.3%, Figure 2B) time points. In comparison, the proportion of neutrophils in the PBS + PTT treated and PBS (as control) groups remained low (PBS + PTT: less than 25%; PBS: less than 31%, Figure 2B). The proportions of macrophages, on the other hand, kept decreasing both in the GC + PTT and GC groups (GC + PTT: from 44.2% at 4 h to 9.7% at 72 h; GC: from 39.2% at 4 h to 17.1% at 72 h, Figure S2B). Conversely, the proportions of macrophages in the PBS + PTT and PBS groups remained at high levels from 4 h to 72 h (PBS + PTT: over 25%; PBS: over 43%, Figure S2B). The proportions of DCs in all treatment groups stayed low from 4 h to 72 h (GC + PTT: less than 5.2%, GC: less than 3.2%, PBS + PTT: less than 5.7%, and PBS: less than 8.1%, Figure S2C). The results indicate that the intratumoral injection of GC induced a rapid infiltration (4 h after treatments) of neutrophils into the treated tumors, and GC combined with or without PTT continued to recruit neutrophils and remained at a high level from the 48-h to the 72-h time point.

Next, we identified the immune cells that ingested GC in the treated tumors. The Rhodamine B labeled GC (GC-RB) was injected into the tumors followed by PTT treatment, 2 h later. Results show that the proportion of neutrophils in the immune cells with GC-RB^+^ (gated by CD45^+^ and RB positive signal; gating strategy is shown in Figure S3A) in the GC + PTT treated tumors increased to a high level as early as 4 h after treatment (~67.3%) and remained high at high levels till 72 h after LIT (Figure 2C, Figure S3B-C). The proportion of macrophages and DCs in the immune cells with GC-RB^+^, meanwhile, stayed at low level (both less than 15%, Figure 2C, Figure S3B-C). Thus, intratumoral GC not only promoted the infiltration of neutrophils, but were also ingested by neutrophils, suggesting that GC plays an immunomodulation antitumor function involving neutrophils.

To evaluate the systemic immune response induced by LIT, changes of cytokines (IL-6, IL-1β, and TNF-α) in the serum of mice bearing CFP-B16 tumors after different treatments were analyzed using enzyme-linked immunosorbent assay (ELISA, Figure 2D-F). As early as 4 h after treatments, the secretion of IL-6 in the serum of the GC and GC + PTT treated mice was 6-fold higher than that of the PBS + PTT treated (or PBS) mice (Figure 2D). Although either PBS + PTT or GC-alone was able to cause an increase in the secretion of cytokines at the early stage (12 h) after treatments, the secretion of cytokines induced by the GC + PTT treatment was significantly higher than those of the other treatments. Quantitative data shows that the secretion of IL-6 in the serum of the GC + PTT mice was 1.7-fold and 28.3-fold higher than that in the GC-alone (or PBS + PTT) and PBS group, respectively; IL-1β in the GC + PTT mice was1.5-fold, 2.1-fold, and 5.6-fold higher than that in the GC-alone, PBS + PTT, and PBS group, respectively; TNF-α in the GC + PTT mice was 2.8-fold, 2.1-fold, and 14-fold higher than that in the GC-alone, PBS + PTT, and PBS group, respectively (Figure 2D-F). Interestingly, the expression of cytokines peaked 12 h after treatments and returned to the normal at 72 h (Figure 2D-F). These results suggest that GC + PTT treatment increased the secretion of cytokines in the serum transiently, which accelerated neutrophil infiltration into the treated tumors further.

Next, we investigated the safety of different treatments on the mice at 24 h and 10 days post treatments. The biochemical analysis showed that at 24 h, compared to the PBS group or GC treated mice, there was some stress responses in PBS + PTT and GC + PTT treated mice, based on the levels of alkaline phosphatase (ALP), alanine aminotransferase (ALT), and aspartate transaminase (AST) of PBS + PTT and GC + PTT treated mice significantly increased (Figure S4A). By comparing GC treated mice with PBS group, and comparing GC + PTT treated mice with PBS + PTT treated mice, the levels of ALP, ALT and AST showed no significant increase. The body weights of the mice had no significant changes in all four groups (Figure S4B). Images of H&E-stained tissue sections showed that there were some vacuoles in the hepatic cells of PBS + PTT and GC + PTT treated mice (Figure S4C). These results suggested that, laser irradiation (PTT), not injection of GC induced the stress response in the livers of treated mice. At 10 days after treatments, the levels of ALP, ALT and AST of PBS + PTT and GC + PTT treated mice returned to the normal (Figure S5A). Meanwhile, compared with PBS + PTT and GC + PTT treated mice, the levels of AST in PBS group and GC treated mice remained elevated (Figure S5A). The body weights of the mice had no significant changes in all four groups (Figure S5B). The morphology of hepatic cells in PBS + PTT and GC + PTT treated mice returned to normal, while histopathologic analysis showed some minor liver injury in PBS and GC treated mice (Figure S5C). All these results suggested that although PTT induced some stress response in the liver as early as 24 h after the treatments, this response was transient and recoverable, and the liver function would return to the normal several days after treatments. The minor liver injury in PBS and GC treated mice was not directly caused by treatments, which might have been induced by tumor growth.

To verify that LIT induced immunogenic death of B16 tumors, we assessed the expression of DAMPs in tumors and TDLNs using Western Blot (WB). The data showed that the expression of HSP70 in the GC + PTT and PBS + PTT treated tumors increased 24 h after treatment, compared with GC treated and PBS tumors (Figure 2G). Remarkably, the expression of HSP70 in TDLNs increased significantly in the GC + PTT mice and increased slightly in the PBS + PTT and GC mice 48 h after treatment (Figure 2H). In the meantime, the expressions of HMGB1 in TDLNs of the GC + PTT, GC and PBS + PTT mice also increased, compared with that of the PBS group (Figure 2H). This result confirmed that LIT promoted the release of DAMPs in the treated tumors and their migration into TDLNs.

As early as 24 h after treatment, a 1.9-fold increase in activated (CD69^+^) CD4^+^ T cells and a 2.5-fold increase in activated (CD69^+^) CD8^+^ T cells were observed in the GC + PTT treated mice, compared to the PBS group (Figure 2I-J). The activation of T cells and the high-level expression of DAMPs in TDLNs promoted by the GC + PTT treatment at early stage (24 h after treatment, Figure 2H-J) is expected to play an important role in triggering an antitumor immune response.

To further evaluate the cellular antitumor immune response induced by LIT, the status of DCs in TDLNs was analyzed after treatment. Notably, the GC + PTT treatment promoted DC maturation efficiently in TDLNs 72 h after treatment. The percentage of mature DCs (CD80^+^ and CD86^+^) increased significantly to 45.6% in the GC + PTT group, compared with PBS group (22.8%), and it was much higher than that in the PBS + PTT (26.9%) and GC group (30.9%), as shown in Figure 2K and Figure S6A-D. These findings suggest that LIT induced release of tumor-specific antigens and DAMPs, which were captured by DCs and transported into TDLNs to stimulate maturation of DCs. These results support our hypothesis that GC + PTT treatment could eliminate primary tumors and promote infiltration of neutrophils that ingest most of the injected GCs, leading to the generation of *in situ* “tumor whole-cell vaccine” and promoting DC activation in TDLNs.

### GC combined with PTT accelerates the generation of long-term immune memory in tumor-bearing mice

**Figure 3:**
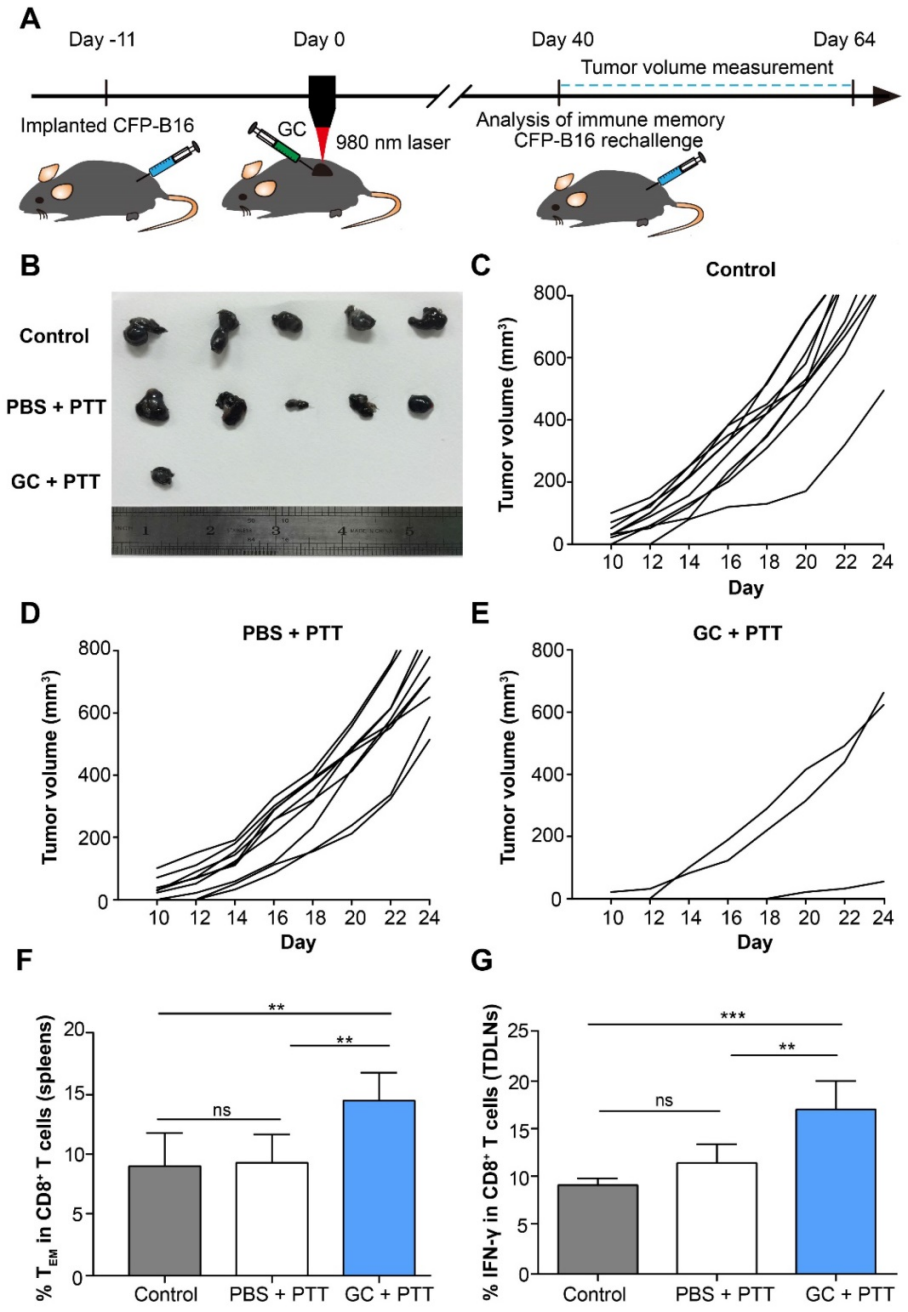
** Long-term tumor resistance induced by LIT. (A)** Schematics of the procedures and timeline of the CFP-B16 tumor rechallenge of successfully treated tumor-bearing mice and immunological assays.** (B)** Tumors collected from mice 24 days after tumor rechallenge. Healthy, age marched C57BL/6 mice were used as control. **(C-E)** Tumor growth curves for mice in different treatment groups (n = 10 mice, two independent experiments) after tumor rechallenge.** (F)** Frequency of effector memory T cells (T_EM_) in the spleens were analyzed (CD8^+^CD62L^-^CD44^+^) on day 40 after tumor treatments. Data are presented as mean ± SD (n = 7-8 mice, three independent experiments). **(G)** IFN-γ secretion by CD8^+^ T cells in TDLNs collected from mice 11 days after tumor rechallenge. Data are presented as mean ± SD (n = 5 mice, two independent experiments). Statistical analysis was performed using the one-way ANOVA test followed by the Bonferroni post-test. * *P* < 0.05, ** *P* < 0.01, *** *P* < 0.001, ns: not significant.

To evaluate the effectiveness of the long-term antitumor immune memory induced by LIT to resist the tumor rechallenge, 2×10^5^ CFP-B16 cells were subcutaneously implanted into the contralateral flank of cured mice 40 days after treatment of the primary tumors. The tumor rechallenge procedures followed the timelines in Figure 3 A. The mice with tumor regression in the GC + PTT and PBS + PTT groups were used for tumor rechallenge (the mice in the PBS and GC groups were all dead before tumor cells rechallenge experiments), and the healthy and age-marched C57BL/6 mice were used as the control. Compared with control and PBS + PTT groups (100% of the rechallenge mice with tumor growth), 70% of the mice in the GC + PTT group were tumor free 24 days after tumor rechallenge (Figure 3B-E). The result indicates that the LIT-elicited antitumor immune response was sustained for a long time and was strong enough to reject the rechallenged tumor.

CD8^+^ effector memory T cells (T_EM_) in the spleens from mice were evaluated 40 days after treatment of the primary tumors. T_EM_ plays an important role in stimulating immediate protective immune responses by producing cytokines, such as IFN-γ, when the same pathogens attack again 33. The flow cytometry data show that the proportion of T_EM_ cells (CD8^+^ CD44^+^ CD62L^-^) in the spleen of the mice treated with GC + PTT (14.7%) was 1.5-fold higher than that in the PBS + PTT (9.5%) or control mice (9.2%), as shown in Figure 3F. Notably, the percentage of T_EM_ in the PBS + PTT and control mice showed no significant difference (Figure 3F). Furthermore, 11 days after tumor rechallenge, the proportion of IFN-γ positive CD8^+^ T cells in TDLNs of the GC + PTT treated mice (17.3%) was 1.5-fold higher than that in the PBS + PTT (11.7%) or control mice (9.3%), as shown in Figure 3G. There was no difference between the proportions of these cells in the PBS + PTT and control mice (Figure 3G). These results suggest that intratumoral injection of GC promoted the generation of a long-term antitumor T cell immune memory in LIT treated mice, and the memory T cells were reactivated successfully when the treated mice were rechallenged with the same type of tumor cells.

### Intravital imaging revealed the motility of endogenous tumor infiltrating lymphocytes in rechallenged tumor microenvironments

**Figure 4:**
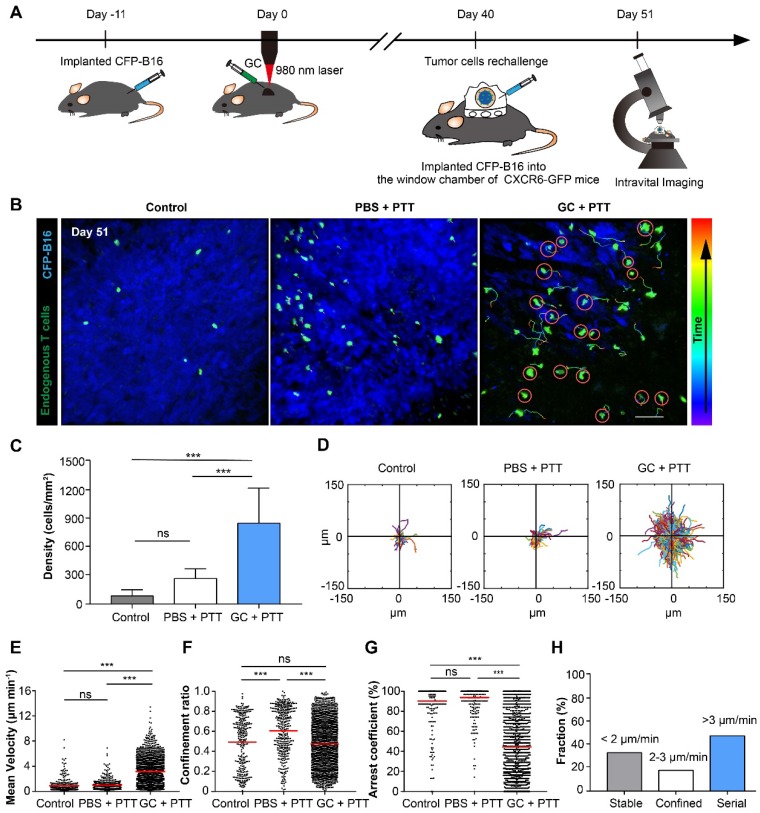
** Migration of endogenous TILs in the tumor microenvironment of CXCR6-GFP mice with CFP-B16 rechallenge. (A)** Schematics of the procedures and timeline of intravital imaging of endogenous TILs in the tumor microenvironment. **(B)** *In vivo* time-lapse images of endogenous GFP^+^ TILs in the CFP-B16 tumor area. Scale bar: 70 μm. **(C)** Quantification of the density of endogenous GFP^+^ TILs on day 11 after CFP-B16 tumor cell implantation. Data are presented as mean ± SD (n = 11-13 fields, from 3-5 mice per group). **(D)** Trajectories of GFP^+^ T cells in different groups, following the alignment of their starting positions. **(E-G)** Scatter plots of the mean velocity **(E)** confinement ratio **(F)** and arrest coefficient **(G)** of GFP^+^ TILs in tumor areas. Each data point represents a single cell, and the red bars indicate mean values. **(H)** Histograms representing the relative fraction of the different classes of interactions between TILs and tumor cells. The data from 5-7 mice, 3 independent experiments were pooled. Statistical analysis was performed using the one-way ANOVA test followed by the Bonferroni post-test, and Kruskal-Wallis test followed by Dunn's multiple comparison tests. * *P* < 0.05, ** *P* < 0.01, ***P < 0.001, ns: not significant.

The movement behavior of tumor infiltrating lymphocytes (TILs) in the tumor microenvironment is crucial for their functionality. To understand the migratory behavior of endogenous TILs in the rechallenged tumors, we used intravital microscopy to observe the dynamic behavior of TILs (GFP^+^) in the tumor areas 11 days after tumor rechallenge (51 days after the treatment of primary tumors), with 2×10^5^ CFP-B16 tumor cells implanted into a skin-fold window chamber on the back of *Cxcr6^+/gfp^* transgenic (CXCR6-GFP) mice. In previous reports, more than 90% of the GFP cells of CXCR6-GFP mice in tumor areas were endogenous TILs 32, 34. The design of the animal experiment is shown in Figure 4A. The imaging data show that the number of TILs (GFP^+^) in the GC + PTT treated mice was 3.2-fold and 10.0-fold higher than that in the PBS + PTT treated mice and control mice, respectively (Figure 4B-C).

We analyzed three parameters quantitatively to describe the motility properties of TILs *in vivo* (Figure 4D-G): the mean velocity, which represents the migratory speed; the confinement ratio, which indicates the ratio of the maximum displacement of each cell from its path length within a given time; the arrest coefficient, which denotes the percentage of time that each cell remained arrested 32, 35. The data show that, compared with the PBS + PTT and control groups, the motility of TILs in the tumor areas of the GC + PTT group were more active (Movie 1), with a high speed and a decreased arrest coefficient (GC + PTT: mean velocity: 3.17 ± 2.05 µm min^-1^ and arrest coefficient: 44 ± 34%, n = 1626 cells, Figure 4E-G). There was no significant difference in the mean velocity and arrest coefficient between the PBS + PTT and control groups (PBS + PTT group: n = 432, *versus* control group: n = 286; mean velocity: 0.99 ± 0.85 *versus* 0.91 ± 1.09 µm min^-1^, arrest coefficient: 91 ± 19% *versus* 90 ± 20%, respectively, Figure 4E-G). The trajectories of TILs in the GC + PTT group were more confined than that in the PBS + PTT and had no significant difference with control (0.47 ± 0.24 in the GC + PTT group, 0.62 ± 0.23 in the PBS + PTT group, and 0.49 ± 0.26 in the control group, respectively, Figure 4D-F). The movement of TILs in the GC + PTT group were most active, causing more endogenous TILs to infiltrate into the tumor areas to search more target tumor cells for elimination.

We further analyzed the interactions between TILs and CFP-B16 tumor cells in the GC + PTT group in more details focusing on the following three types of interactions. According to previous studies 36, 37, the first type, called “stable”, corresponds to TILs arrested to closely contact with tumor cells (mean velocity < 2 μm min^-1^). The second, called “confined”, corresponds to TILs not completely arrested but that moved around tumor cells (mean velocity: 2-3 μm min^-1^). And the third, called “serial”, corresponds to TILs interacting with tumor cells transiently or not interacting with any tumor cells (mean velocity > 3 μm min^-1^). In the GC + PTT group, almost half of TILs remained in stable or confined interactions with CFP-B16 tumor cells (circled in red, Figure 4B). The proportion of TILs with mean velocity less than 2 μm min^-1^ was 33.6%, between 2-3 μm min^-1^ was 18.1%, and higher than 3 μm min^-1^ was 48.3% (Figure 4H). We concluded that, although the mobility of TILs in the GC + PTT group was most active, more than half of the TILs were in close contact with tumor cells with a stable or confined interaction. The observed stable and confined interactions between TILs and tumor cells indicated that the TILs scanned and recognized specific antigens of tumor cells, and then efficiently eliminated them 32, 36, 38. This result suggested that the TILs in the GC + PTT mice had strong abilities to eliminate tumor cells, as evidenced by the smaller tumor area in the GC + PTT mice than that in the other groups (Figure 4B).

### LIT modified the component of endogenous TILs by increasing CTLs and decreasing Tregs

The *ex vivo* analysis of endogenous GFP^+^ TILs in the distant tumors of different groups was performed on CFP-B16 tumors using flow cytometry, 11 days after tumor rechallenge. The data show that the percentage of activated cells (CD69^+^) in the endogenous GFP cells of the tumors of the GC + PTT group was 2.1-fold and 5.2-fold higher than that in the PBS + PTT group and control group (65.6% in the GC + PTT group, *versus* 31.9% in the PBS + PTT group, and 12.5% in the control group, Figure S7A-B). In the GC + PTT group, the percentage of CD8^+^ T cells of the GFP^+^ cells of the tumors were higher than those in the other two groups (60.2% in the GC + PTT group, *versus* 42.9% in the PBS + PTT group, and 43.3% in the control group, Figure S7C). Most CD8^+^ T cells (CTLs) of GFP^+^ cells were activated based on the expression of activation marker CD69 in the GC + PTT group (76.8%, Figure S7D), which was 3.1-fold and 5.3-fold higher than that in the PBS + PTT group (24.7%) and control group (14.4%, Figure S7D). There was no significant difference in the percentage of CD4^+^ T cells of the GFP^+^ cells between different groups (Figure S7E). Importantly, the proportion of Tregs in the CD4^+^ T cells of GFP^+^ cells decreased significantly in the GC + PTT group, compared with that in the other two groups (13.4% in the GC + PTT group, *versus* 36.2% in the PBS + PTT group, and 50.3% in the control group, Figure S7F). Tregs played an important role in the immunosuppression of the tumor microenvironment. Therefore, it is beneficial to reduce Tregs in the tumor area to enhance the anti-tumor efficiency of activated CTLs. These data indicated that GC + PTT treatment could promote the activation of CTLs and decrease Tregs in the TILs.

### LIT-induced long-term immune memory inhibited lung metastasis

**Figure 5:**
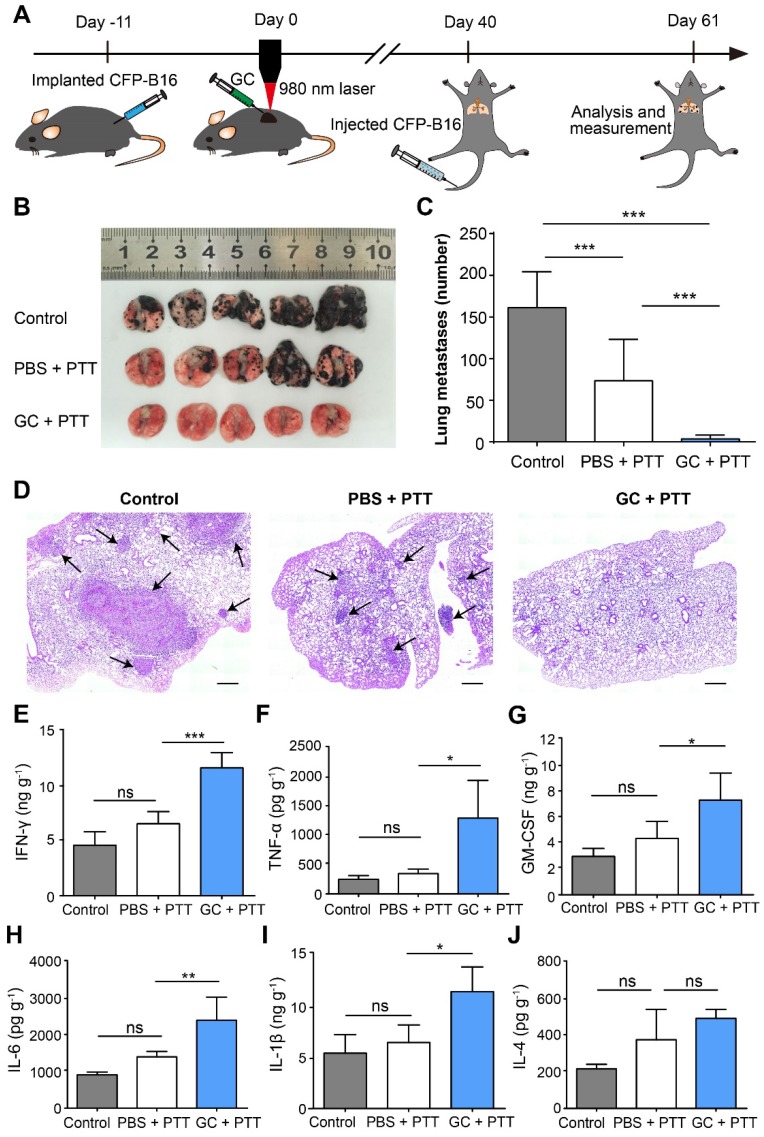
** Inhibition of lung metastasis by LIT. (A)** Schematics of the procedures and timeline of LIT in the inhibition of CFP-B16 tumor lung metastasis. **(B)** Tumor nodules in the lungs. Lungs collected from mice of different groups 21 days after 2×10^5^ CFP-B16 tumor cells were injected through tail vein. **(C)** Number of metastases in the lungs of different mice groups. Data are presented as mean ± SD (n = 9-12 mice, two independent experiments). **(D)** H&E staining of lung tissues of different mice groups. Scale bar: 500 μm. **(E-J)** Cytokine levels (IFN-γ, IL-1 β, GM-CSF, IL-6, TNF-α, and IL-4) in the lungs collected from mice in different groups, and were analyzed using ELISA. Data are presented as mean ± SD (n = 3-5 mice, two independent experiments). Statistical analysis was performed using the one-way ANOVA test followed by the Bonferroni post-test. * *P* < 0.05, ** *P* < 0.01, *** *P* < 0.001, ns: not significant.

To determine the effectiveness of antitumor immune memory induced by LIT on inhibition of lung metastasis, the survival mice were rechallenged through tail vein (*i.v.*) injections of 2×10^5^ CFP-B16 cells, 40 days after the initial treatment of the primary tumors, to generate the lung metastasis, as shown in Figure 5A. The mice were sacrificed at the designated time (day 21) to analyze the nodes of lung metastases. The direct observation (Figure 5B-C) and histochemical (Figure 5D) analysis showed that lung metastases were significantly inhibited by the GC + PTT treatment (3 nodes/lung, *P* < 0.001), compared with those in the other two groups (PBS + PTT group: 73 nodes/lung and control group: 161 nodes/lung, Figure 5 C).

Using ELISA, we also analyzed the secretion of cytokines (IFN-γ, TNF-α, GM-CSF, IL-1β, IL-6, and IL-4) in the lungs of different groups on the same day the lungs were extracted from the mice with metastases. As shown in Figure 5E-J, in the GC + PTT group, the expression of cytokines with antitumor functions, including IFN-γ, TNF-α, and GM-CSF was 1.8-fold, 3.7-fold, and 1.7-fold, respectively, higher than that in the PBS + PTT group (Figure 5E-G). Furthermore, the expression of cytokines IL-1β and IL-6 in the GC + PTT group was both 1.7-fold higher than that in the PBS + PTT group (Figure 5H-I), whereas, the expression of the cytokine IL-4 with a pro-tumor function was not significantly different between all the groups (Figure 5J). These results indicate that the GC + PTT treatment induced a long-term immune memory to resist the rechallenge of tumor cells and inhibited lung metastases.

### PD-1 blockade enhanced the effect of LIT on the distant secondary B16 tumors

**Figure 6:**
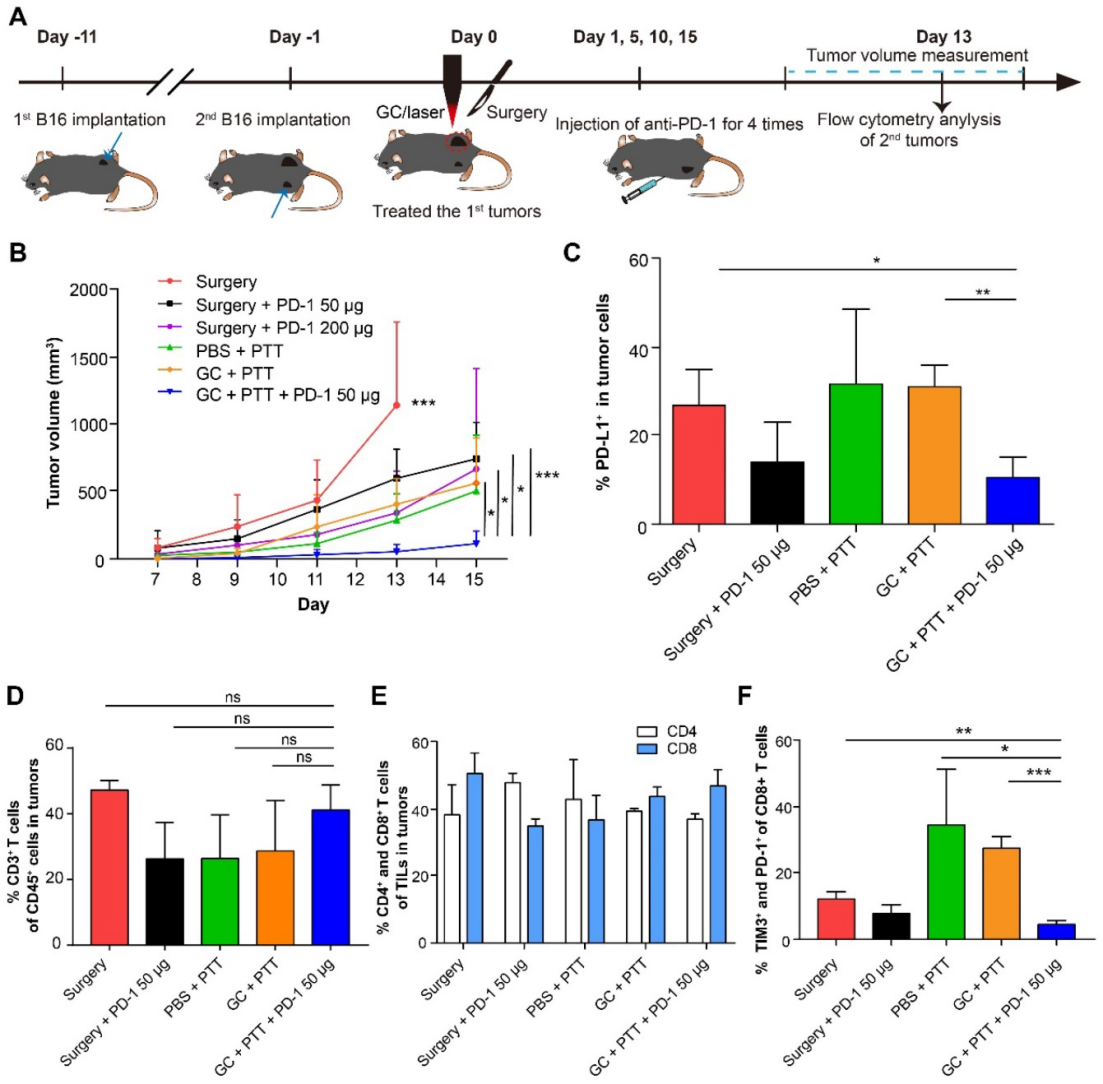
** Synergistic inhibitory effect of LIT combined anti-PD-1 for distant secondary B16 tumors. (A)** Schematics of the procedures and timeline of LIT combined with anti-PD-1 to treat primary and secondary B16 tumors. **(B)** Tumor growth curves (both flanks) in different groups. Data are presented as mean ± SD (n = 6-7 mice). **(C)** Percentage of PD-L1^+^ cells in the secondary tumor cells 13 days after different treatments of the first tumors.** (D)** Percentage of CD3^+^ T cells (TILs) in the CD45^+^ immune cells in the secondary tumors after various treatments.** (E)** Percentage of CD4^+^ and CD8^+^ T cells in TILs from **(D)**. **(F)** Percentage of TIM3^+^ and PD-1^+^ cells in CD8^+^ T cells from **(E)**. Data are presented as mean ± SD (n = 3-4 mice). Statistical analysis was performed using the unpaired *t*-test, or Mann-Whitney test (nonparametric). * *P* < 0.05, ** *P* < 0.01, *** *P* < 0.001, ns: not significant.

To improve efficacy of distant secondary B16 tumors, we treated the mice bearing the secondary tumors with combination therapy: the primary tumor was treated with LIT first, followed by intravenous injection of a PD-1 antibody four times. The experimental design is shown in Figure 6A. Briefly, 2 × 10^5^ B16 cells were implanted subcutaneously on the contralateral flank (as the secondary tumors) of the mice with primary tumors (defined as Day -1). One day later, the primary tumors were treated with LIT (defined as Day 0) followed by four anti-PD-1 administration (on Day 1, 5, 10 and 15). The volumes of both primary and secondary tumors in the mice were measured. Compared with the control groups, the mice that received the LIT + 50 μg anti-PD-1 therapy showed effective tumor inhibition, with their tumor volumes only 20% of that in the CG + PTT group (*P* < 0.05), and 17% of that in the surgery + 200 μg anti-PD-1 group (*P* < 0.05, Figure 6B).

To further understand the mechanism of antitumor immune effect induced by LIT + anti-PD-1 treatment, both tumor cells and immune cells in the distant secondary tumors were analyzed using flow cytometry 14 days after they were implanted. Results from the anti-PD-1 treatments showed that the percentage of tumor cells expressing PD-L1 decreased noticeably to ~14% (both in the surgery + anti-PD-1 50 μg and LIT + anti-PD-1 50 μg groups). In other groups without anti-PD-1 treatments, percentages of tumor cells expressing PD-L1 were about 30% (Figure 6C). Although the percentages of CD3^+^ (TILs), CD4^+^, and CD8^+^ T cells in the tumors had no significant changes in all groups (Figure 6D and E), the proportion of exhausting CD8^+^ T cells (TIM3^+^ and PD-1^+^, which represents dysfunction of T cell) decreased significantly (from 35% in the PBS + PTT group to 4.56% in the LIT + anti-PD-1 50 μg groups, Figure 6F). These results suggest that anti-PD-1 treatment could synergize with LIT-induced antitumor immunity by reducing the expression of PD-L1 on the surface of B16 tumor cells efficiently and decreasing the proportion of exhausting CD8^+^ T cells (reversing dysfunction of CTLs). These results indicate that while LIT has limited effect on the secondary B16 tumors, but LIT combined with a checkpoint inhibitor (anti-PD-1) could have synergistic effects on both tumor cells and CTLs, potentially achieving an optimized anti-tumor effect on secondary tumors.

### Timeline of LIT mediated antitumor immune response for primary, secondary, and rechallenged and metastatic melanoma

**Figure 7:**
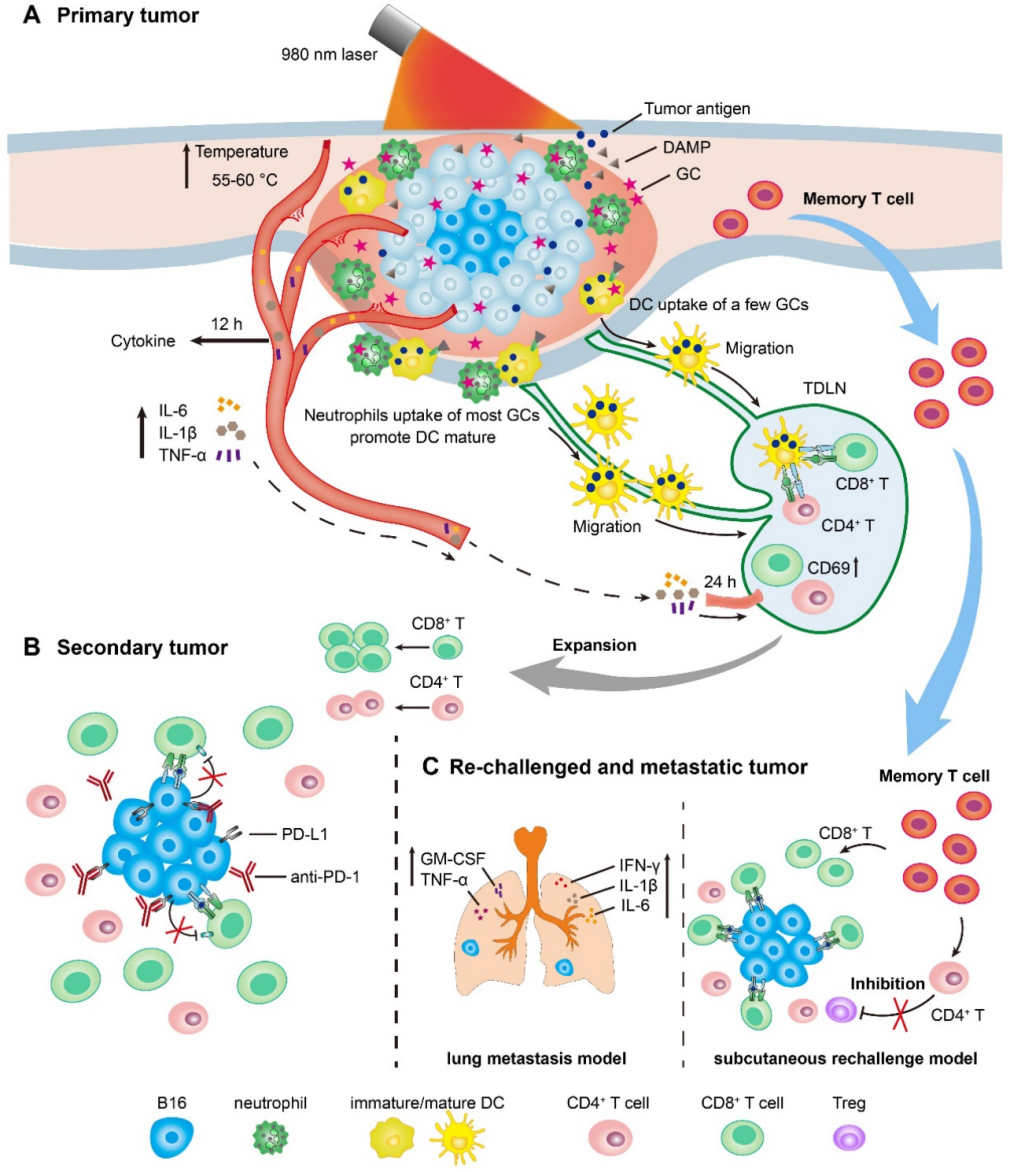
** Schematics of LIT mediated antitumor immune response for primary, secondary, and re-challenged and metastatic melanoma. (A)** Schematic illustration of laser immunotherapy (LIT) for subcutaneous primary tumor and the timelines of the immune response in the tumor, serum and TDLNs. **(B)** Schematic of the treatment of secondary tumors by the combination of LIT and the anti-PD-1 antibody. **(C)** Schematic of the antitumor mechanism of LIT in the subcutaneous re-challenged tumors and lung metastasis.

We have drawn the timeline of immune events in the treated primary tumors, TDLNs, secondary tumors, and rechallenged tumors, according to the immunological analysis of antitumor immune response induced by LIT (Figure 7). For the primary tumor treatment (Figure 7A), GC injection-promoted neutrophils quickly migrated into the primary tumor areas. Neutrophils ingested most GC, while DCs and macrophages ingested only a small portion of GCs. LIT promoted the secretion of various cytokines as early as 12 h after treatment, and the increased secretion of cytokines induced the activation of T cells in TDLNs transiently 24 h after treatments. The release of DAMPs in the treated tumors presented the immunologic death of tumor cells and promoted the maturation of DCs in TDLNs 72 h after treatments. For the secondary tumor treatment, 1 day after the administration of LIT (Figure 7B), the combination of LIT with anti-PD-1 showed synergistic antitumor effects by decreasing the expression of PD-L1 on the surface of tumor cells and reversing dysfunction of CTLs. Because of the generation of T memory cells, 40 days after the treatment of primary tumors, strong tumor inhibition effects on both subcutaneous rechallenged tumor and lung metastasis (Figure 7C) were generated in the LIT-treated mice. In the subcutaneous tumor rechallenge model, it appeared that LIT caused the changes in the components of endogenous TILs, with increased CTLs and decreased Tregs in the rechallenged tumors. In the lung metastasis model, LIT led to an increase in the secretion of important cytokines, including IFN-γ, IL-1β, IL-6, TNF-α, and GM-CSF to inhibit metastasis (Figure 7C).

## Discussion

In this study, we used the combination of photothermal ablation and intratumor administration of GC, a semisynthetic biopolymer with remarkable immune-stimulating properties, to induce a neutrophil-involved synergistic antitumor immune response that could eliminate both treated primary tumors and untreated distant metastasis, as well as prevent tumor recurrence. When combined with the checkpoint inhibitor PD-1 blockade, the enhanced immunological response controlled the distal untreated tumors efficiently, by decreasing the expression of PD-L1 on tumor cells and reversing dysfunction of CTLs.

We used intravital imaging to observe the movement behavior of endogenous T cells in the tumor rechallenge sites (Figure 4). To the best of our knowledge, our study is the first to focus on the tumor rechallenge sites to dynamically monitor the interactions between endogenous TILs and tumor cells by intravital imaging. Compared with the control and the PBS + PTT groups, the mobility of endogenous T cells in the GC + PTT treated mice were most active (Figure 4D-G, Movie 1), which is different from findings of previous reports 32, 34. Most endogenous GFP^+^ T cells in previous studies are CD8^+^ T cells 32, 34. In our study, the activated CD8^+^ T cells (CD69^+^) increased in the GC + PTT treated mice (compared with the other two groups, Figure S7C-D), and the proportions of CD4^+^ T cells of endogenous TILs showed no significant changes among the three groups (Figure S7E). The movement of endogenous TILs in the GC + PTT group were more active than that in the other two groups, allowing TILs to be more robust in their search for and destruction of target tumor cells. Although the mobility of TILs in the GC + PTT group was enhanced overall, more than half of the TILs maintained in arrested and confined movements, indicating that a sufficient number of TILs interacted with tumor cells to eliminate them.

Neutrophils are the first responders to infection and injury 39. Most disruptions of tumor microenvironment could trigger infiltration of neutrophils, such as PTT, injection of agents, cytokines, necrosis, and DAMPs. However, the roles of the infiltrating neutrophils in the tumor microenvironment are still poorly understood. Tumor associated neutrophils (TANs) reportedly have both antitumoral and protumoral effects 40. Some studies have suggested that TANs carry out the immunosuppression function in tumor microenvironments and promote tumor growth and metastasis 41. Other studies, meanwhile, have reported that the infiltration of neutrophils into tumors is important for an antigen-specific antitumor immune response 42 and a radiation-induced antitumor immune response 43. We found that the intratumoral injection of GC induced neutrophil infiltration, which played a key role in the maturation of DCs and the generation of immune memory. Based on the protocol of LIT in our previous studies, we changed the therapeutic schedule to intratumoral injection of GC first, followed by laser irradiation 2 h later. The intratumoral GC induced a rapid infiltration of neutrophils into tumors as early as 4 h after injection. Whether the tumor was treated with or without laser irradiation, the proportion of neutrophils in the CD45^+^ immune cells increased to a high level (more than 60%) (Figure 2B). Interestingly, most of the intratumoral GC was ingested by neutrophils, and a small amount was taken up by macrophages and DCs (Figure 2C).

Although the subsequent laser irradiation decreased the proportion of neutrophils transiently, it did not affect the uptake of GC by neutrophils (Figure 2C). It is worth noting that, the significant increase of neutrophils infiltrating into the treated tumors is uniquely induced by GC. The possible reason is that, GC (N-dihydrogalactochitosan) is a derivative of chitosan, and neutrophil was reported to be able to identify the chitosan (e.g. 81.6% and 80.6% deacetylated chitosan) 44 and to have a chemotactic migration to the chitosan when it implanted into mice 30. Importantly, the maturation of DCs in TDLNs increased significantly 72 h after the treatment of LIT (Figure 2K and Figure S6A-D). The previous studies reported that, glycosylation-dependent cellular interactions between neutrophils and immature DCs through β_2_-integrin Mac-1 and C-type lectin DC-SIGN (the adhesion receptor DC-specific intracellular associated molecular (ICAM)-3 grabbing non-integrin) promoted the maturation of DC by neutrophils producing TNF-α 45-47. Thus, the intratumoral GC activated DCs in three possible ways: (1) GC, mainly taken up by neutrophils (Figure 2C), mediated the glycosylation-dependent interactions between neutrophils and DCs to activate DCs and promote the migration of mature DCs into TDLNs; (2) GC increased the secretion of TNF-α in serum, which then contributed to the neutrophil mediated maturation of DCs 45, 47; (3) DCs ingested some GCs (Figure 2C and Figure S3B) and, in combination with LIT-induced immunogenic tumor cell death and the release of DAMPs (Figure 2G), promoted the maturation of DCs. The maturation of DC enhanced the systematic antitumor immune response and induced the long-term antitumor T cell immune memory 48.

T cell immune memory is an important feature of cancer vaccine and can prevent tumor recurrence. Our results indicate that GC as an immunostimulant is necessary for generation of an antitumor immune memory, with the whole-cell tumor vaccine produced by the *in situ* PTT treatment. We analyzed the antitumor immune memory using a subcutaneous tumor rechallenge model (Figure 3) and lung metastasis model 40 days after the treatment of LIT on the primary tumors (Figure 5). Before the rechallenge, the proportions of effective memory T cells (T_EM_) in the mice with LIT-induced primary tumor regression were analyzed by flow cytometry. Compared to the control group and PBS + PTT group, the proportion of T_EM_ in the GC + PTT group was significantly higher (Figure 3F). Correspondingly, the GC + PTT treatment obviously inhibited the rechallenged tumor growth (Figure 3B-E) and lung metastasis (Figure 5). Meanwhile, both the expression of IFN-γ in the CD8^+^ T cells of TDLNs (Figure 3G) and the secretion of some important cytokines in the lungs (Figure 5E-J) increased in the GC + PTT group. Consequently, our data demonstrate that LIT-induced neutrophil-involved whole-cell tumor vaccine promoted the generation of antitumor T cell immune memory, which could be converted efficiently into a specific antitumor immune response against rechallenged tumor cells.

Recently, nanotechnology-based photoimmunotherapy has made significant advances in cancer therapy. The versatile characteristics, such as carrying and releasing drugs by nanoparticles 19, 20, 49-52 and penetrating cells 20, 53, 54, make nanomaterials promising agents for phototherapy mediated immune responses. However, the clinical applications of most nanomaterials are still facing challenges. Nanomaterials alone usually could not induce sufficient antitumor immune responses when combined with phototherapy; antibodies and/or immunostimulant/immunoadjuvant have to be conjugated to construct a complex nanoplatform, which involves several components 17, 55, 56. Our LIT approach only involves GC and 980 nm laser, which possesses promising potential in clinical applications.

LIT appears to be an easy-to-operate, low-toxicity method that generates a whole-cell tumor vaccine *in situ* and triggers a strong, long-term antitumor immune response. The whole-cell tumor vaccine contains the entire spectrum of tumor-specific and common antigens, allowing the host immune system to select the target antigens and recognize the tumor cells with specificity 13, 14. The local treatment with PTT could lead to tumor regression, release whole-cell tumor antigens and DAMPs (Figure 2G), and even lead to long-term survival (Figure 1D and F). HSP 70 and HMGB 1 are the most important DAMPs, which directly promote the activation of APCs and antigen presentation 57-59. PTT alone was insufficient to induce a strong systemic antitumor immune response or generate a long-term antitumor immune memory, in order to prevent the tumor relapse and metastasis, as shown in Figure 3B and D. Injection of GC induced a significant infiltration of neutrophils into tumors (Figure 2 B-C); however, it had no effect on tumor elimination, release of tumor antigens and DAMPs, or generation of antitumor immune memory. Thus, the PTT-initiated tumor antigens/DAMPs release and GC-induced neutrophil infiltration are the two key elements for generation of long-term antitumor immune memory. It is worth noting, that the whole-cell tumor vaccine has a drawback: it has the potential to induce high expression of PD-L1 in tumor cells, helping them to escape the elimination by immune cells 15.

To further enhance the curative effect of GC + PTT, particularly against secondary tumors, we combined GC + PTT with a low-dose (50 μg) of checkpoint blockade PD-1 antibody (Figure 6). This combination therapy achieved an optimized antitumor effect on secondary tumors and provided a valuable therapy strategy for its clinical application. On the one hand, LIT helped to reduce the dosage of anti-PD-1, in comparison with surgery + anti-PD-1 200 μg group, hence decreasing its immunotoxicity, which is the main side effect of the checkpoint inhibitors 7, 10, 11. Furthermore, even at a low-dose, PD-1 antibody enhanced the antitumor effect of LIT by reducing the expression of PD-L1 on tumor cells and reversing dysfunction of CTLs in the tumors. It has been reported that, PD-L1 on the tumor cells and PD-1 on the T cells can influence each other 60. Specifically, the PD-1 checkpoint inhibitor can effectively reduce the PD-1 expression of T cells, and influence the status of tumor cells, such as reducing the expression of PD-L1 on the tumor cells, facilitating the attack and elimination of tumor cells by T cells. In addition, other researchers reported that the PD-1 antibody treatment could reduce the expression of PD-L1 on the tumor cells 61, 62.

## Conclusion

In summary, we have presented a personalized immunotherapy by combining an intratumoral injection of an immunostimulant (GC) with local PTT. This combination therapy produced a whole-cell tumor vaccine and triggered neutrophil-involved systemic antitumor immunity. The LIT strategy is applicable in solid malignant tumors for the generation of long-term immune memory to prevent tumor recurrence and metastasis. Furthermore, LIT synergized with a checkpoint inhibitor to achieve an improved antitumor efficacy. Our study demonstrates that LIT-elicited neutrophil infiltration and whole-cell vaccine generation enhanced the antitumor immune response and long-term immune memory, showing promises as a potential personalized cancer immunotherapy strategy for recurrent and metastatic melanoma.

## Materials and Methods

### Mice

C57BL/6 female mice were obtained from the Hunan Slack King of Laboratory Animal Co., Ltd (Hunan, China). B6.129P2-Cxcr6tm1Litt/J (*Cxcr6^+/gfp^*, JAX: 005693) mice were derived from breeding pairs that were obtained originally from the Jackson Laboratory (Bar Harbor, ME, USA). All the mice were bred and maintained in a specific pathogen-free (SPF) barrier facility at the Animal Center of Wuhan National Laboratory for Optoelectronics. All animal studies were approved by the Hubei Provincial Animal Care and Use Committee and followed the experimental guidelines of the Animal Experimentation Ethics Committee of the Huazhong University of Science and Technology.

### Tumor cells

B16 melanoma cells were purchased from Boshide Biology Ltd. China. The CFP-B16 tumor cell line was established in our lab 32. All cell lines were treated with 25 μg/ml Plasmocin™ (InvivoGene, Toulouse, France) for at least two weeks and were mycoplasma-negative as determined by MycoProbe Mycoplasma Detection Kit (R&D Systems, Minneapolis, MN, USA). The cell lines were authenticated using the Cell Line Authentication Service of short-tandem-repeat (STR) profiling carried out by Beijing Microread Genetics Co., Ltd. (Beijing, China). The cells were cultured in RPMI-1640 medium (HyClone, Beijing, China) containing 10% fetal bovine serum (FBS, HyClone).

### *In vivo* tumor growth and LIT administration

Mice were divided randomly into groups. For the first-round of tumor implantation, B16 cells or CFP-B16 cells (5×10^5^) suspended in PBS were injected subcutaneously into the right flank of each female C57BL/6 mouse. After 11 days, primary tumors were injected intratumorally with GC or PBS, followed 2 h later by treatment of tumors with a 980 nm laser (Changchun New Industries Optoelectronics Technology Co., Ltd, Changchun, China) radiation (the diameter of laser spot is 11mm) with a power intensity of 1 W/cm^2^ for 10 min. At the same time, temperature changes were recorded by an infrared thermal camera (VarioCAM, InfraTec, Dresden, Germany). For the second-round of tumor implantation, which was conducted 40 days after the first treatments, 2×10^5^ CFP-B16 cells suspended in PBS were injected subcutaneously into the contralateral (left) flank of mice with primary tumor regression. The tumor volume was calculated using the following formula: V = L (length) × W (width) ×H (height)/2 32. To establish lung metastases, 2×10^5^ CFP-B16 cells were administered intravenously via infusion at the tail vein into the mice with primary tumor regression, 40 days after the first treatments. Lung metastases appeared as black nodules on the surface of the lungs and were counted under a microscope (Changfang, Shanghai, China).

### Western Blot Analysis

To detect expression of DAMPs (HSP 70, HMGB1) in tumor tissues and TDLNs, Western Blots were carried out using supernatants, separated on 10% SDS-PAGE gels, and transferred to polyvinylidene difluoride membranes (Millipore). The membranes were blocked for 2 h with 5% skimmed milk powder diluted with Tris-buffered saline (TBS) with 0.1% Triton X-100 (Sigma-Aldrich, St. Louis, MO, USA), and incubated with the primary antibodies of HSP70 (1:2000, ab181606, Abcam, Cambridge, United Kingdom), HMGB1 (1:10000, ab79823, Abcam) and β-actin (1:2000, 60008-1-Ig, Proteintech, Rosemont, IL, USA) for 12 h at 4 °C. Membranes were then incubated with a solution with horseradish peroxidase-conjugated secondary antibody (1:2000, SA00001-9, Proteintech) for 1 h at 37 °C. An enhanced chemiluminescence reagent (Beyotime biotech, Shanghai, China) was used to detect the signals on the membrane. Indicated protein levels were analyzed by normalizing to those of the internal controls (β-actin).

### Cytokine detection

Serum samples were isolated from mice after treatments and diluted for analysis. Lung samples were lysed with RIPA solution (Beyotime biotech). Tumor necrosis factor (TNF-α), interferon gamma (IFN-γ), IL-1β, IL-4, IL-6, and GM-CSF were analyzed using ELISA kits (all from Biolegend, San Diego, CA, USA) according to the product manuals.

### Flow cytometry

To study the immune cells in the primary and secondary tumors, tumor tissues were digested with 1 mg/ml collagenase IV (Worthington, Lakewood, NJ, USA) and 0.1 mg/ml DNase (Sigma-Aldrich) for 45 min at 37 °C. For the detection of intracellular IFN-γ, cells were stimulated with a Brefeldin-A solution and a Cell Activation Cocktail (all from Biolegend) at 37 °C for 4 h. For the detection of Foxp3, cells were permeabilized and fixed using a transcription factor staining buffer set (Biolegend). Cells were infused with a Fixable Viability Dye eFluor™ 506 (Thermo Fisher, Carlsbad, CA, USA) for 30 min at 4 °C and then stained with the following antibodies. CD45-Alexa Flour 488 (Clone 30F-11, Cat# 103122), CD11b-PE/Cy7 (Clone M1/70, Cat# 101216), CD11c-Alexa Flour 647 (Clone N418, Cat# 117313), CD11c-APC/Cy7 (Clone N418, Cat# 117324), Ly6c-PerCP/Cy5.5 (Clone HK1.4, Cat# 128012), Ly6G-APC/Cy7 (Clone 1A8, Cat# 127624), CD80-PE (Clone 16-10A1, Cat# 104070), CD86-APC (Clone GL-1, Cat# 105011), CD3-PE (Clone 145-2C11, Cat# 100308), CD3-APC/Cy7 (Clone 145-2C11, Cat# 100308), CD4-APC (Clone GK1.5, Cat# 100421), CD4-Alexa Flour 647 (Clone GK1.5, Cat# 100424), CD4-PE/Cy7 (Clone GK1.5, Cat# 100422), CD69-PE/Cy7 (Clone H1.2F3, Cat# 104512), IFN-λ-PE (Clone XMG1.2, Cat# 505808), Foxp3-Alexa Flour 647 (Clone MF23, Cat# 560402), PD-L1-PE (clone 10F.9G2, Cat# 124307), CD62L-PE/Cy7 (Clone MEL-14, Cat# 100706), and CD44-PerCP-Cy5.5(Clone IM7, Cat# 103032), all from Biolegend. F4/80-BV421 (Clone T45-2342, Cat# 565411), CD3-BV650 (Clone 145-2C11, Cat# 564378), CD8-PerCP-Cy5.5 (Clone 53-6.7, Cat# 551162), CD8-PE/Cy7 (Clone 53-6.7, Cat# 552877), CD8-PE (Clone 53-6.7, Cat# 553032), CD4-BV421 (Clone GK1.5, Cat# 562891), CD45-BV605 (Clone 30-F11, Cat# 563053), all from BD Biosciences (San Jose, CA, USA). All the cells were examined using a CytoFLEX S Flow Cytometer (Beckman Coulter, Brea, CA, USA). The data were analyzed using FlowJo software (FlowJo, BD).

### The preparation of the skin-fold window chamber and injection of CFP-B16 tumor cells

The window chamber on the mouse was prepared as previously described 32, 35. Briefly, 40 days after the primary tumors were treated, the CXCR6-GFP mice with primary tumor regression were anesthetized by *i.p.* injecting a mix of ketamine (100 mg kg-1, Sigma-Aldrich, USA) and xylazine (10 mg kg-1, Sigma-Aldrich, USA) and positioned on a warmer plate at 37 °C (Thermo Plate, TOKAI HIT, Japan). A pair of Titanium window frames (APJ Trading Co., Inc., Ventura, CA, USA) was implanted on the back of the mouse. One day later, CFP-B16 tumor cells (2 × 10^5^ resuspended in 20 μL PBS) were injected into the chamber and at a site near the major vessel of the mouse. The entire surgical process was carried out under sterile conditions to avoid infection. The mice received Tolfedine via *i.p.* injection (16.25 mg kg^-1^, Vétoquinol, Québec, Canada) once a day for three days to relieve pain associated with surgery and inflammation.

### Intravital imaging of the tumor microenvironment

The mice with window chambers were anesthetized by inhaling 1.0-3.0% isoflurane in oxygen flow through a Matrx VMS small animal anesthesia machine (Midmark, Dayton, OH, USA). The window chamber was fixed on a warm plate (Thermo Plate) using a custom-made holder and then fastened to the microscope stage. Intravital imaging was obtained with the large-field imaging function on a motorized stage using an A1R MP+ System (Nikon, Tokyo, Japan). The images were captured using the 20× objective (N.A. 0.75, Nikon). A confocal laser scanning microscope was used to simultaneously image the CFP-B16 cells (405 nm excitation, 400-500 nm emission), and CXCR6-GFP cells (488 nm excitation, 500-550 nm emission).

### Histological analysis

The hearts, livers, spleens, lungs and kidneys were extracted from the tumor-bearing mice with different treatments, and the lungs with metastases were extracted from the tumor rechallenged mice and then fixed in 4% paraformaldehyde for 24-48 h at 4 °C. The organs were embedded in paraffin, sectioned, and stained with hematoxylin and eosin (H&E). The H&E experiments were performed by Biossci Company (Biossci Biotechnology Co. Ltd, Wuhan, China). Images were obtained using a Nikon Ni-E microscope (Nikon, Tokyo, Japan).

### Blood Biochemical Analyses

Blood samples were collected from the treated mice at 24 h and 10 days after treatments. The blood biochemical analyses were performed using a biochemical analyzer (SPOTCHEM EZ SP-4430, Arkray Inc., Kyoto, Japan).

### Data analysis

The movements of immune cells were tracked and analyzed by Imaris 7.6 (Bitplane AG, Zurich, Switzerland) software. The mean velocity, arrest coefficient, and confinement ratio were also calculated using the Imaris 7.6. The results of mean velocity, the arrest coefficient, and the confinement ratio were calculated as previously described 32, 63.

### Statistical analysis

Statistical analysis was performed using GraphPad Prism 7 (GraphPad Software, Inc., La Jolla, CA, USA). For comparisons of three or more groups, the one-way ANOVA test followed by Bonferroni post-test or the Kruskal-Wallis test followed by Dunn's multiple comparison tests was applied. For comparisons of two groups, the two-tailed unpaired *t*-test or Mann-Whitney test was used. The statistical analysis is described in each figure legend. Differences between or among groups are denoted as ns for not significant, * for* P* < 0.05, ** for *P* < 0.01, and *** for *P* < 0.001.

## Supplementary Material

Supplementary figures and movie legend.

Supplementary movie.

## Abbreviations

ACT: adoptive cell therapy
APCs: antigen presenting cells
CFP: cyan fluorescent protein
CTLA-4: cytotoxic T-lymphocyte-associated protein 4
CTLs: cytotoxic T cells
DAMPs: damage-associated molecular patterns
DCs: dendritic cells
DC-SIGN: the adhesion receptor dendritic cell-specific intracellular adhesion molecule-3-grabbing non-integrin
ELISA: enzyme-linked immunosorbent assay
FBS: fetal bovine serum
GC: N-dihydrogalactochitosan
GFP: green fluorescent protein
GM-CSF: granulocyte-macrophage colony-stimulating factor
H&E: hematoxylin and eosin
HMGB1: high mobility group box 1
HSP70: heat shock protein 70
IFN-γ: interferon-γ
IL-1β: interleukin-1β
IL-4: interleukin 4
IL-6: interleukin 6
LIT: laser immunotherapy
PBS: phosphate buffered saline
PD-1: programmed cell death-1
PD-L1: programmed death ligand-1
PTT: photothermal therapy
RB: rhodamine B
RIPA: Radioimmunoprecipitation Assay
RPMI-1640: Roswell park memorial institute-1640
SDS-PAGE: sodium dodecyl sulfate polyacrylamide gel electrophoresis
STR: short-tandem-repeat
TANs: tumor-associated neutrophils
TBS: tris-buffered saline
TDLNs: tumor-draining lymph nodes
T_EM_: effector memory T cells
TILs: tumor-infiltrating lymphocytes
TIM3: T-cell immunoglobulin and mucin domain-3
TNF-α: tumor necrosis factor-α
WB: western blot

## References

[1] Lo JA, Fisher DE (2014). The melanoma revolution: From UV carcinogenesis to a new era in therapeutics. Science.

[2] Schadendorf D, Fisher DE, Garbe C, Gershenwald JE, Grob JJ, Halpern A (2015). Melanoma. Nat Rev Dis Primers.

[3] Tarhini A, Ghate SR, Ionescu-Ittu R, Manceur AM, Ndife B, Jacques P (2018). Postsurgical treatment landscape and economic burden of locoregional and distant recurrence in patients with operable nonmetastatic melanoma. Melanoma Res.

[4] Bombelli FB, Webster CA, Moncrieff M, Sherwood V (2014). The scope of nanoparticle therapies for future metastatic melanoma treatment. Lancet Oncol.

[5] Sanmamed MF, Chen L (2018). A Paradigm Shift in Cancer Immunotherapy: From Enhancement to Normalization. Cell.

[6] Li X, Song W, Shao C, Shi Y, Han W (2019). Emerging predictors of the response to the blockade of immune checkpoints in cancer therapy. Cell Mol Immunol.

[7] Boutros C, Tarhini A, Routier E, Lambotte O, Ladurie FL, Carbonnel F (2016). Safety profiles of anti-CTLA-4 and anti-PD-1 antibodies alone and in combination. Nat Rev Clin Oncol.

[8] June CH, Warshauer JT, Bluestone JA (2017). Is autoimmunity the Achilles' heel of cancer immunotherapy?. Nat Med.

[9] Lin C, Zhang J (2019). Chimeric antigen receptor engineered innate immune cells in cancer immunotherapy. Sci China Life Sci.

[10] Moslehi JJ, Salem JE, Sosman JA, Lebrun-Vignes B, Johnson DB (2018). Increased reporting of fatal immune checkpoint inhibitor-associated myocarditis. Lancet.

[11] Godwin JL, Jaggi S, Sirisena I, Sharda P, Rao AD, Mehra R (2017). Nivolumab-induced autoimmune diabetes mellitus presenting as diabetic ketoacidosis in a patient with metastatic lung cancer.

[12] Tanyi JL, Bobisse S, Ophir E, Tuyaerts S, Roberti A, Genolet R (2018). Personalized cancer vaccine effectively mobilizes antitumor T cell immunity in ovarian cancer.

[13] Kaiser J (2017). Personalized tumor vaccines keep cancer in check. Science.

[14] Chiang CLL, Coukos G, Kandalaft LE (2015). Whole Tumor Antigen Vaccines: Where Are We?. Vaccines.

[15] Okazaki T, Honjo T (2006). The PD-1-PD-L pathway in immunological tolerance. Trends Immunol.

[16] Chu KF, Dupuy DE (2014). Thermal ablation of tumours: biological mechanisms and advances in therapy. Nat Rev Cancer.

[17] Nakajima T, Sano K, Choyke PL, Kobayashi H (2013). Improving the Efficacy of Photoimmunotherapy (PIT) using a Cocktail of Antibody Conjugates in a Multiple Antigen Tumor Model. Theranostics.

[18] Fujimoto S, Muguruma N, Okamoto K, Kurihara T, Sato Y, Miyamoto Y (2018). A Novel Theranostic Combination of Near-infrared Fluorescence Imaging and Laser Irradiation Targeting c-KIT for Gastrointestinal Stromal Tumors. Theranostics.

[19] Chen Q, Xu L, Liang C, Wang C, Peng R, Liu Z (2016). Photothermal therapy with immune-adjuvant nanoparticles together with checkpoint blockade for effective cancer immunotherapy. Nat Commun.

[20] Wang TT, Wang DG, Yu HJ, Feng B, Zhou FY, Zhang HW (2018). A cancer vaccine-mediated postoperative immunotherapy for recurrent and metastatic tumors. Nat Commun.

[21] Chen WR, Adams RL, Carubelli R, Nordquist RE (1997). Laser-photosensitizer assisted immunotherapy: a novel modality for cancer treatment. Cancer Lett.

[22] Chen WR, Singhal AK, Liu H, Nordquist RE (2001). Antitumor immunity induced by laser immunotherapy and its adoptive transfer. Cancer Res.

[23] El-Hussein A, Lam SSK, Raker J, Chen WR, Hamblin MR (2017). N-dihydrogalactochitosan as a potent immune activator for dendritic cells. J Biomed Mater Res A.

[24] Zhou F, Yang J, Zhang Y, Liu M, Lang ML, Li M (2018). Local Phototherapy Synergizes with Immunoadjuvant for Treatment of Pancreatic Cancer through Induced Immunogenic Tumor Vaccine. Clin Cancer Res.

[25] Zhou FF, Li XS, Song S, Acquaviva JT, Wolf RF, Howard EW (2013). Anti-Tumor Responses Induced by Laser Irradiation and Immunological Stimulation Using a Mouse Mammary Tumor Model. J Innov Opt Health Sci.

[26] Luo M, Shi L, Zhang FH, Zhou FF, Zhang LL, Wang B (2018). Laser immunotherapy for cutaneous squamous cell carcinoma with optimal thermal effects to enhance tumour immunogenicity. Int J Hyperthermia.

[27] Li X, Naylor MF, Le H, Nordquist RE, Teague TK, Howard CA (2010). Clinical effects of in situ photoimmunotherapy on late-stage melanoma patients A preliminary study. Cancer Biol Ther.

[28] Li XS, Ferrel GL, Guerra MC, Hode T, Lunn JA, Adalsteinsson O (2011). Preliminary safety and efficacy results of laser immunotherapy for the treatment of metastatic breast cancer patients. Photochem Photobiol Sci.

[29] Naylor MF, Zhou FF, Geister BV, Nordquist RE, Li XS, Chen WR (2017). Treatment of advanced melanoma with laser immunotherapy and ipilimumab. J Biophotonics.

[30] Hoemann CD, Fong D (2017). Immunological responses to chitosan for biomedical applications. Jennings JA, Bumgardner JD, editors.

[31] Moran HBT, Turley JL, Andersson M, Lavelle EC (2018). Immunomodulatory properties of chitosan polymers. Biomaterials.

[32] Qi S, Li H, Lu L, Qi Z, Liu L, Chen L (2016). Long-term intravital imaging of the multicolor-coded tumor microenvironment during combination immunotherapy.

[33] Mueller SN, Mackay LK (2016). Tissue-resident memory T cells: local specialists in immune defence. Nat Rev Immunol.

[34] Ruocco MG, Pilones KA, Kawashima N, Cammer M, Huang J, Babb JS (2012). Suppressing T cell motility induced by anti-CTLA-4 monotherapy improves antitumor effects. J Clin Invest.

[35] Li H, Qi S, Jin H, Qi Z, Zhang Z, Fu L (2015). Zigzag Generalized Levy Walk: the In Vivo Search Strategy of Immunocytes. Theranostics.

[36] Boissonnas A, Fetler L, Zeelenberg IS, Hugues S, Amigorena S (2007). In vivo imaging of cytotoxic T cell infiltration and elimination of a solid tumor. J Exp Med.

[37] Michonneau D, Sagoo P, Breart B, Garcia Z, Celli S, Bousso P (2016). The PD-1 Axis Enforces an Anatomical Segregation of CTL Activity that Creates Tumor Niches after Allogeneic Hematopoietic Stem Cell Transplantation. Immunity.

[38] Qi S, Shi H, Liu L, Zhou L, Zhang Z (2019). Dynamic visualization of the whole process of cytotoxic T lymphocytes killing B16 tumor cells in vitro. J Biomed Opt.

[39] Singel KL, Segal BH (2016). Neutrophils in the tumor microenvironment: trying to heal the wound that cannot heal. Immunol Rev.

[40] Coffelt SB, Wellenstein MD, de Visser KE (2016). Neutrophils in cancer: neutral no more. Nat Rev Cancer.

[41] Wculek SK, Malanchi I (2015). Neutrophils support lung colonization of metastasis-initiating breast cancer cells. Nature.

[42] Yang F, Liu S, Liu X, Liu L, Luo M, Qi S (2016). In Vivo Visualization of Tumor Antigen-containing Microparticles Generated in Fluorescent-protein-elicited Immunity. Theranostics.

[43] Takeshima T, Pop LM, Laine A, Iyengar P, Vitetta ES, Hannan R (2016). Key role for neutrophils in radiation-induced antitumor immune responses: Potentiation with G-CSF. Proc Natl Acad Sci U S A.

[44] Hoemann CD, Chen GP, Marchand C, Tran-Khanh N, Thibault M, Chevrier A (2010). Scaffold-Guided Subchondral Bone Repair Implication of Neutrophils and Alternatively Activated Arginase-1+Macrophages. Am J Sports Med.

[45] van Gisbergen KPJM, Geijtenbeek TBH, van Kooyk Y (2005). Close encounters of neutrophils and DCs. Trends Immunol.

[46] Boudaly S (2009). Activation of dendritic cells by polymorphonuclear neutrophils. Frontiers in Bioscience-Landmark.

[47] van Gisbergen KPJM, Sanchez-Hernandez M, Geijtenbeek TBH, van Kooyk Y (2005). Neutrophils mediate immune modulation of dendritic cells through glycosylation-dependent interactions between Mac-1 and DC-SIGN. J Exp Med.

[48] Wculek SK, Cueto FJ, Mujal AM, Melero I, Krummel MF, Sancho D (2019). Dendritic cells in cancer immunology and immunotherapy.

[49] Chen W, Qin M, Chen X, Wang Q, Zhang Z, Sun X (2018). Combining photothermal therapy and immunotherapy against melanoma by polydopamine-coated Al2O3 nanoparticles. Theranostics.

[50] Ou W, Jiang L, Thapa RK, Soe ZC, Poudel K, Chang JH (2018). Combination of NIR therapy and regulatory T cell modulation using layer-by-layer hybrid nanoparticles for effective cancer photoimmunotherapy. Theranostics.

[51] Ye WY, Li H, Li X, Fan XL, Jin Q, Ji J (2019). mRNA Guided Intracellular Self-Assembly of DNA-Gold Nanoparticle Conjugates as a Precise Trigger to Up-Regulate Cell Apoptosis and Activate Photothermal Therapy. Bioconjug Chem.

[52] Huang SG, Fong CI, Xu MZ, Han BN, Yuan Z, Zhao Q (2019). Nano-loaded natural killer cells as carriers of indocyanine green for synergetic cancer immunotherapy and phototherapy.

[53] Zhou BQ, Song J, Wang M, Wang X, Wang JL, Howard EW (2018). BSA-bioinspired gold nanorods loaded with immunoadjuvant for the treatment of melanoma by combined photothermal therapy and immunotherapy. Nanoscale.

[54] Nam J, Son S, Ochyl LJ, Kuai R, Schwendeman A, Moon JJ (2018). Chemo-photothermal therapy combination elicits anti-tumor immunity against advanced metastatic cancer.

[55] Sato K, Nagaya T, Choyke PL, Kobayashi H (2015). Near infrared photoimmunotherapy in the treatment of pleural disseminated NSCLC: preclinical experience. Theranostics.

[56] Jing H, Weidensteiner C, Reichardt W, Gaedicke S, Zhu XK, Grosu AL (2016). Imaging and Selective Elimination of Glioblastoma Stem Cells with Theranostic Near-Infrared-Labeled CD133-Specific Antibodies. Theranostics.

[57] Murshid A, Gong J, Calderwood SK (2012). The role of heat shock proteins in antigen cross presentation. Front Immunol.

[58] Garg AD, Nowis D, Golab J, Vandenabeele P, Krysko DV, Agostinis P (2010). Immunogenic cell death, DAMPs and anticancer therapeutics: An emerging amalgamation. Biochimica Et Biophysica Acta-Reviews on Cancer.

[59] Ochando J, Ordikhani F, Boros P, Jordan S (2019). The innate immune response to allotransplants: mechanisms and therapeutic potentials. Cell Mol Immunol.

[60] Chen L, Han X (2015). Anti-PD-1/PD-L1 therapy of human cancer: past, present, and future. J Clin Invest.

[61] Taube JM, Klein A, Brahmer JR, Xu H, Pan X, Kim JH (2014). Association of PD-1, PD-1 ligands, and other features of the tumor immune microenvironment with response to anti-PD-1 therapy. Clin Cancer Res.

[62] Rice AE, Latchman YE, Balint JP, Lee JH, Gabitzsch ES, Jones FR (2015). An HPV-E6/E7 immunotherapy plus PD-1 checkpoint inhibition results in tumor regression and reduction in PD-L1 expression. Cancer Gene Ther.

[63] Cahalan MD, Parker I (2008). Choreography of cell motility and interaction dynamics imaged by two-photon microscopy in lymphoid organs. Annu Rev Immunol.

