# Supplementary material for: Neutrophil infiltration and whole-cell vaccine elicited by N-dihydrogalactochitosan combined with NIR phototherapy to enhance antitumor immune response and T cell immune memory
Source: Theranostics. 2020 Jan 1;10(4):1814–32. doi: 10.7150/thno.38515 (PMC6993227; doi:10.7150/thno.38515)
Supplement: Supplementary file 1 — Supplementary figures and movie legend. [file thnov10p1814s1.pdf]

Supplementary Materials

Neutrophil infiltration and whole-cell vaccine elicited by N-dihydrogalactochitosan combined with NIR phototherapy to enhance antitumor immune response and T cell immune memory

Shuhong Qi,[1,2]† Lisen Lu,[1,2]† Feifan Zhou,[3] Yuzhou Chen,[1,2] Mengli Xu,[1,2]

Lu Chen,[1,2]Xiang Yu,[1,2] Wei R. Chen[3]* and Zhihong Zhang[1,2]*

[1]Britton Chance Center for Biomedical Photonics, Wuhan National Laboratory for Optoelectronics-Huazhong University of Science and Technology, Wuhan, Hubei 430074, China

[2]MoE Key Laboratory for Biomedical Photonics, Collaborative Innovation Center for Biomedical Engineering, School of Engineering Sciences, Huazhong University of Science and Technology, Wuhan, Hubei 430074, China

[3]Biophotonics Research Laboratory, Center for Interdisciplinary Biomedical Education and Research, College of Mathematics and Science, University of Central Oklahoma, Edmond, Oklahoma 73034, United States

† These authors contributed equally to this work

* Correspondence: Zhihong Zhang, czyzzh@mail.hust.edu.cn; Wei R. Chen, WChen@uco.edu

Address: Room G304, Britton Chance Center for Biomedical Photonics, Wuhan National Laboratory for Optoelectronics-Huazhong University of Science and Technology, Wuhan, Hubei 430074, China

Fax: +86-27-87792034; Tel: +86-27-87792033;

Room 208 Howell Hall, College of Mathematics and Science, University of Central Oklahoma, Edmond, Oklahoma 73034, United States

Fax: +1-405-974-5147; Tel: +1-405-974-3824;

Zhihong Zhang (czyzzh@mail.hust.edu.cn) and Wei R. Chen (WChen@uco.edu) are the corresponding authors for communication with the Editorial and Production offices.

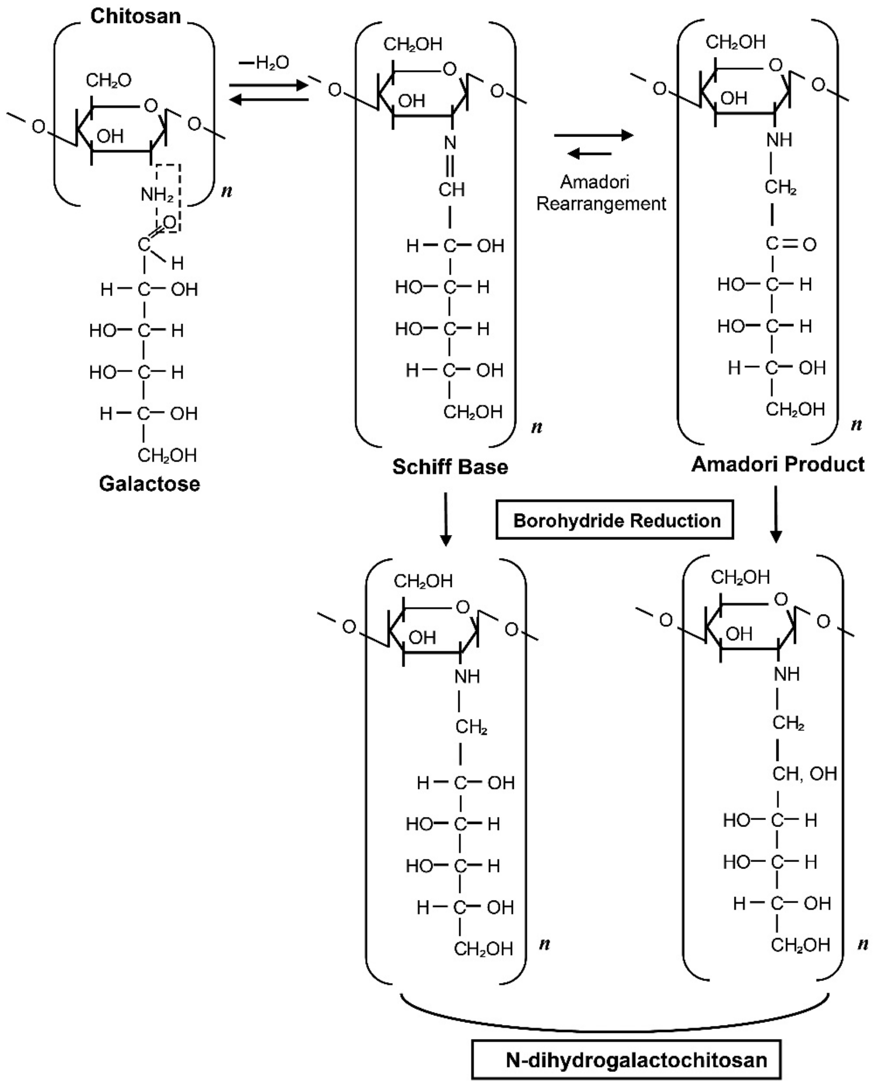

Figure S1. The structure and synthesis process of GC.

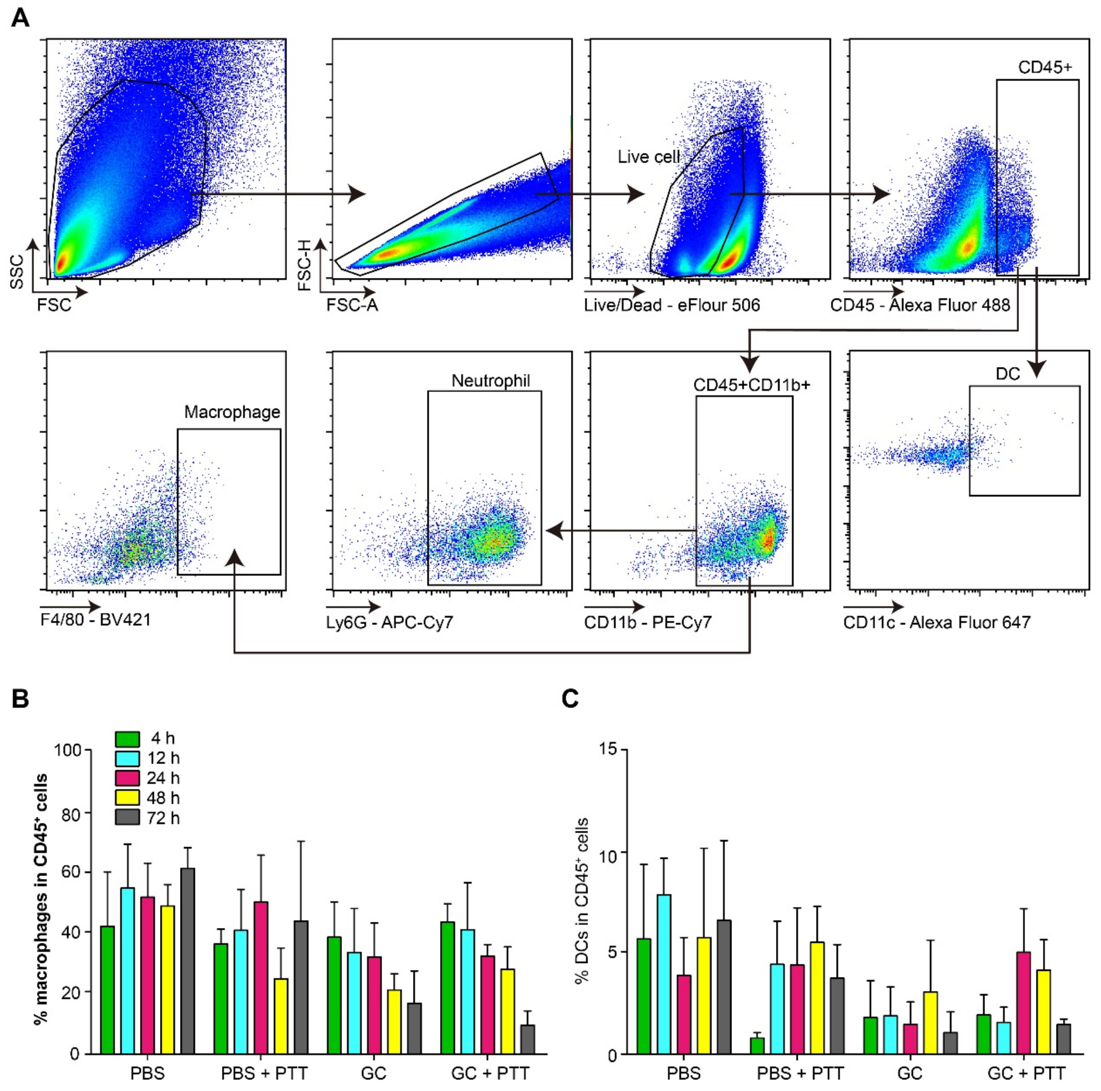

Figure S2. Analysis of neutrophils, macrophages, and DCs in immune cells (CD45^{+}) of various treated tumors at different times using flow cytometry. (A) Gating strategy for neutrophils, macrophages, and DCs in immune cells (CD45^{+}) in tumors. **(B and C)** Proportion of macrophages **(B)** and DCs **(C)** in immune cells (CD45^{+}) of various treated tumors at different times. Data are presented as mean ± SD (n = 3-5 mice, two independent experiments).

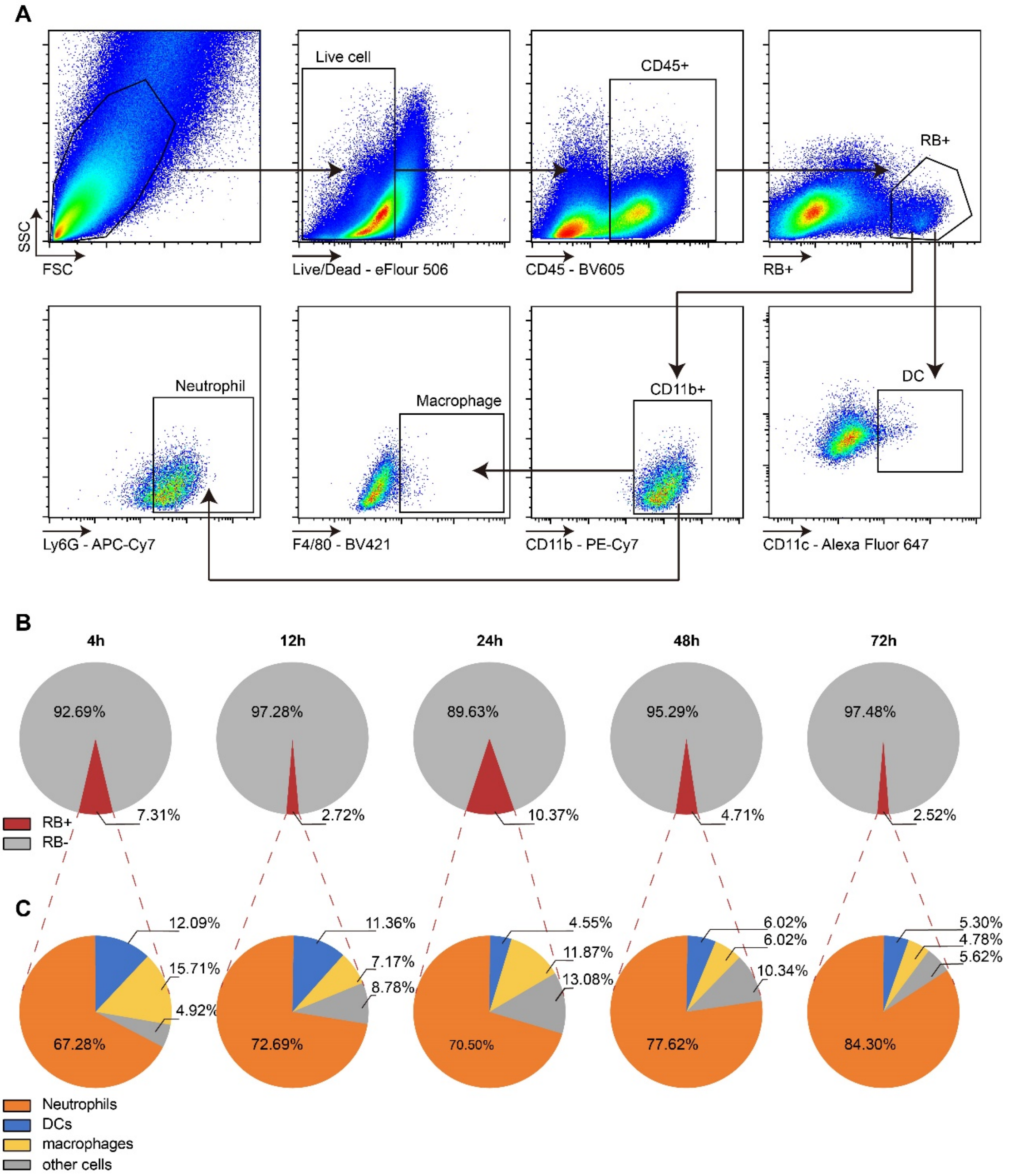

Figure S3. Analysis of neutrophils, macrophages, and DCs in RB signal positive immune cells ($CD45^+RB^+$) in the GC-RB + PTT treated tumors at different times using flow cytometry. (A) Gating strategy for neutrophils, macrophages, and DCs in RB signal positive immune cells ($CD45^+RB^+$) in the GC-RB + PTT treated tumors. **(B)** Proportion of RB positive signal cells in the $CD45^+$ immune cells at different times. **(C)** Proportion of neutrophils, macrophages, and DCs in the RB signal positive immune cells at different times.

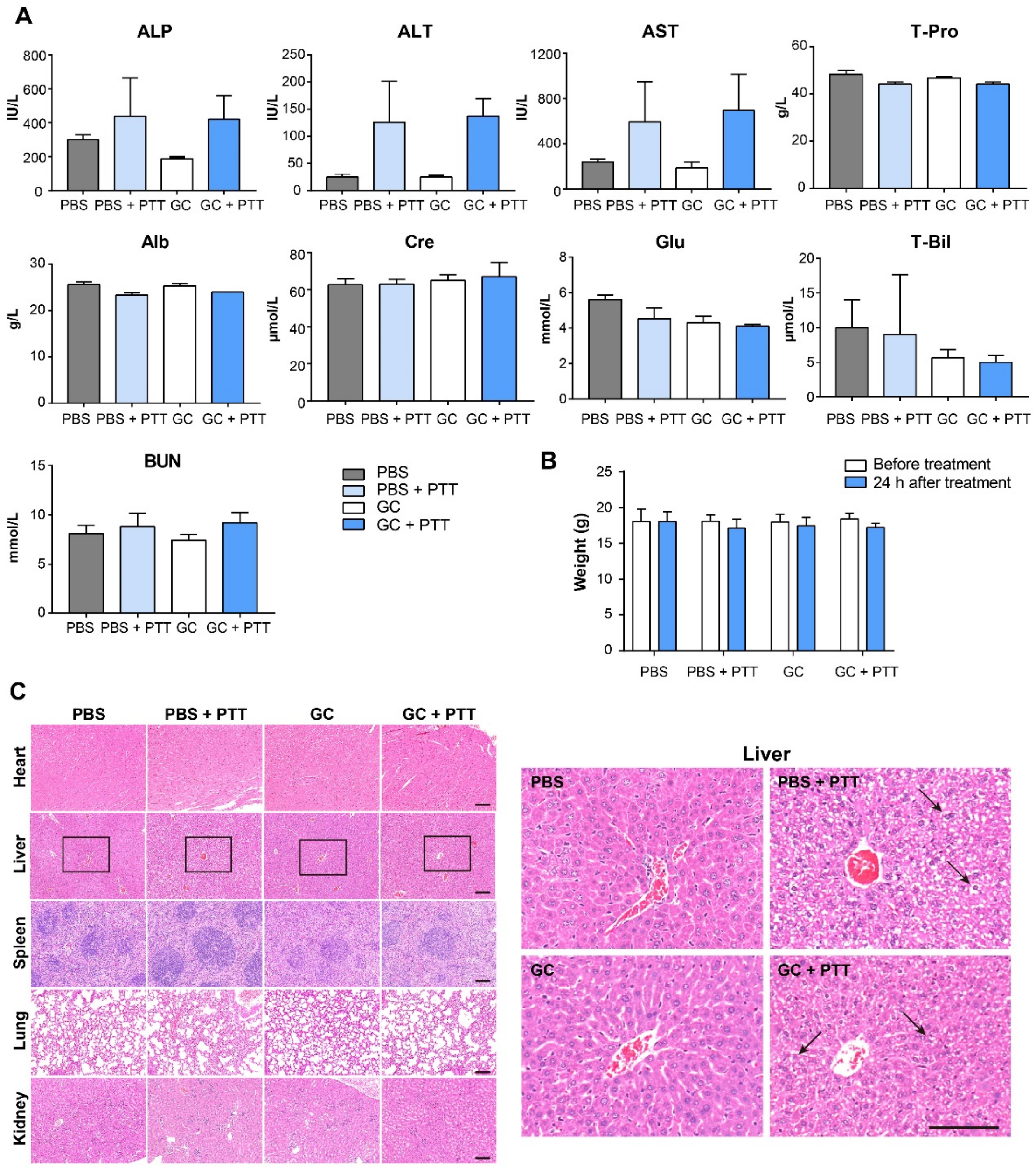

Figure S4. Evaluation of side effects on the mice at 24 h after treatments. (A) Biochemical analysis of blood from treated mice at 24 h. Data are presented as mean ± SD (n = 3 mice). ALP, alkaline phosphatase; ALT, alanine aminotransferase; AST, aspartate transaminase; T-Pro, Total Protein; Alb, albumin; Cre, creatinine; Glu, Glucose; T-Bil, Total Bilirubin; BUN, blood urea nitrogen. **(B)** Body weight changes of mice before treatments and 24 h after treatments. Data are presented as mean ± SD (n = 5 mice). **(C)** Histopathologic analysis of H&E-stained tissue sections of hearts, livers, spleens, lungs, and kidneys from mice at 24 h after treatments. Right panel is zoom images of the black box in the livers. Arrows indicated the vacuoles in the hepatic cells of livers. Scale bar: 100 μm.

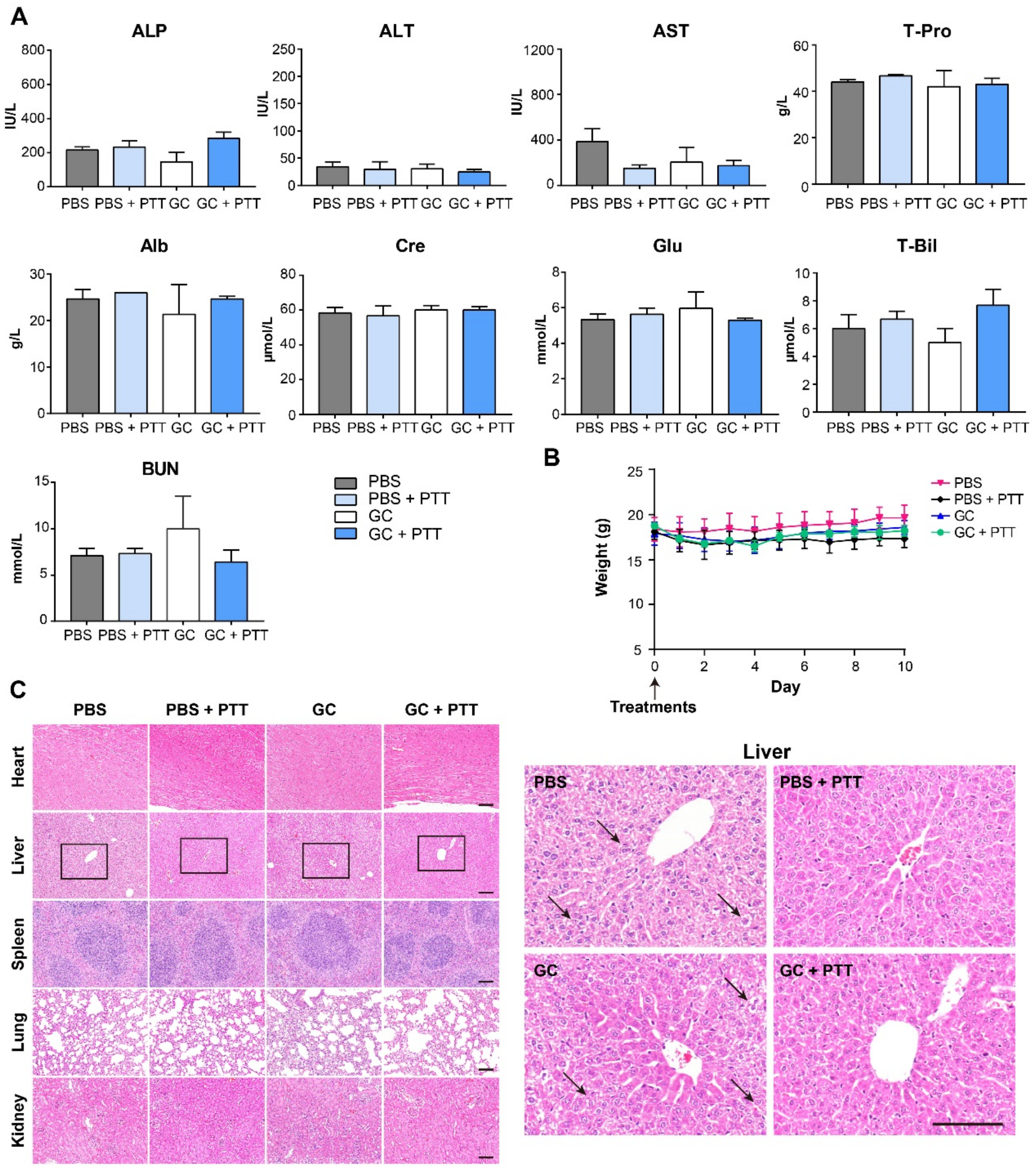

Figure S5. **Evaluation of side effects on the mice at 10 days after treatments. (A)** Biochemical analysis of blood from treated mice at 10 days. Data are presented as mean ± SD (n = 3 mice). **(B)** Body weight changes of treated mice. Data are presented as mean ± SD (n = 5-6 mice). **(C)** Histopathologic analysis of H&E-stained tissue sections of hearts, livers, spleens, lungs, and kidneys from mice at 10 days after treatments. Right panel is the zoom images of black box in the livers. Arrows indicated the vacuoles in the hepatic cells of livers. Scale bar: 100 μm.

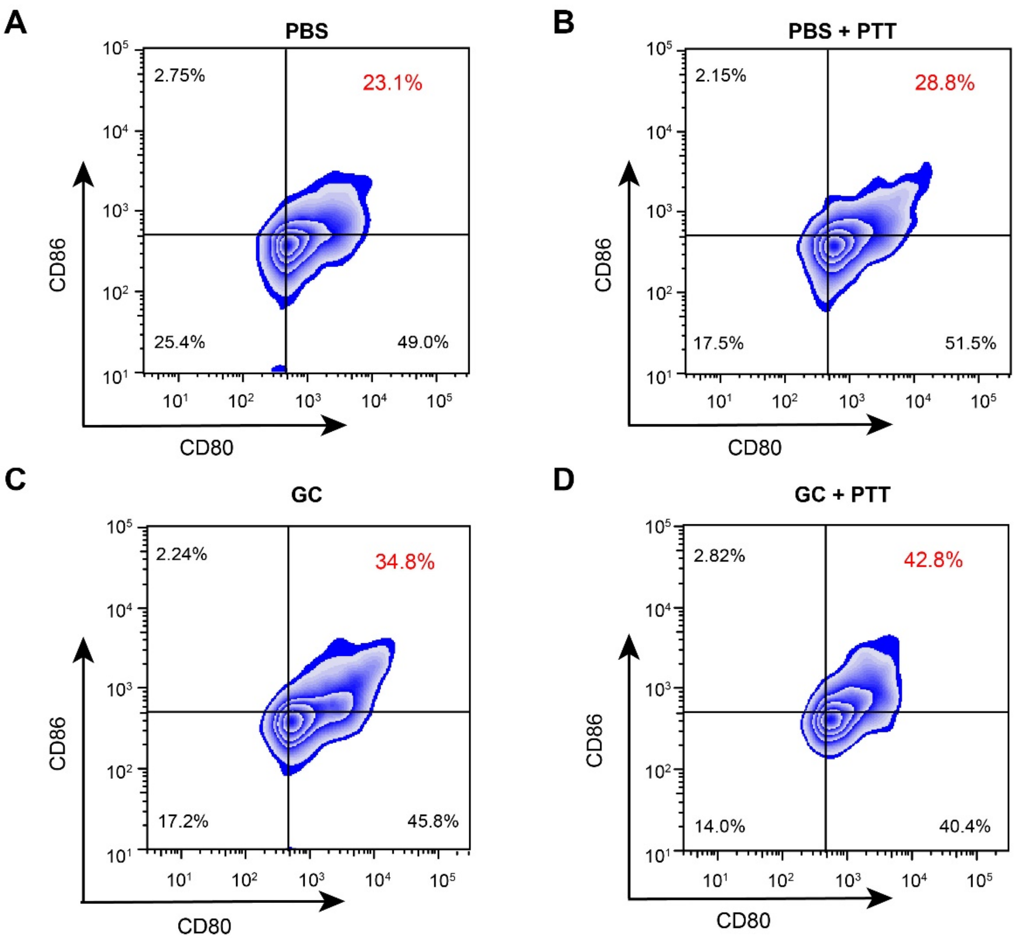

Figure S6. **Frequency of mature DCs (CD11c⁺CD80⁺CD86⁺) in TDLNs 72 h after various treatments. (A-D)** DC maturation induced by PBS **(A)**, PBS + PTT **(B)**, GC **(C)**, and GC + PTT **(D)** in TDLNs of CFP-B16 tumor bearing mice. Cells in the TDLNs were collected 72 h after various treatments for analysis by flow cytometry after staining with CD11c, CD80, and CD86 antibodies.

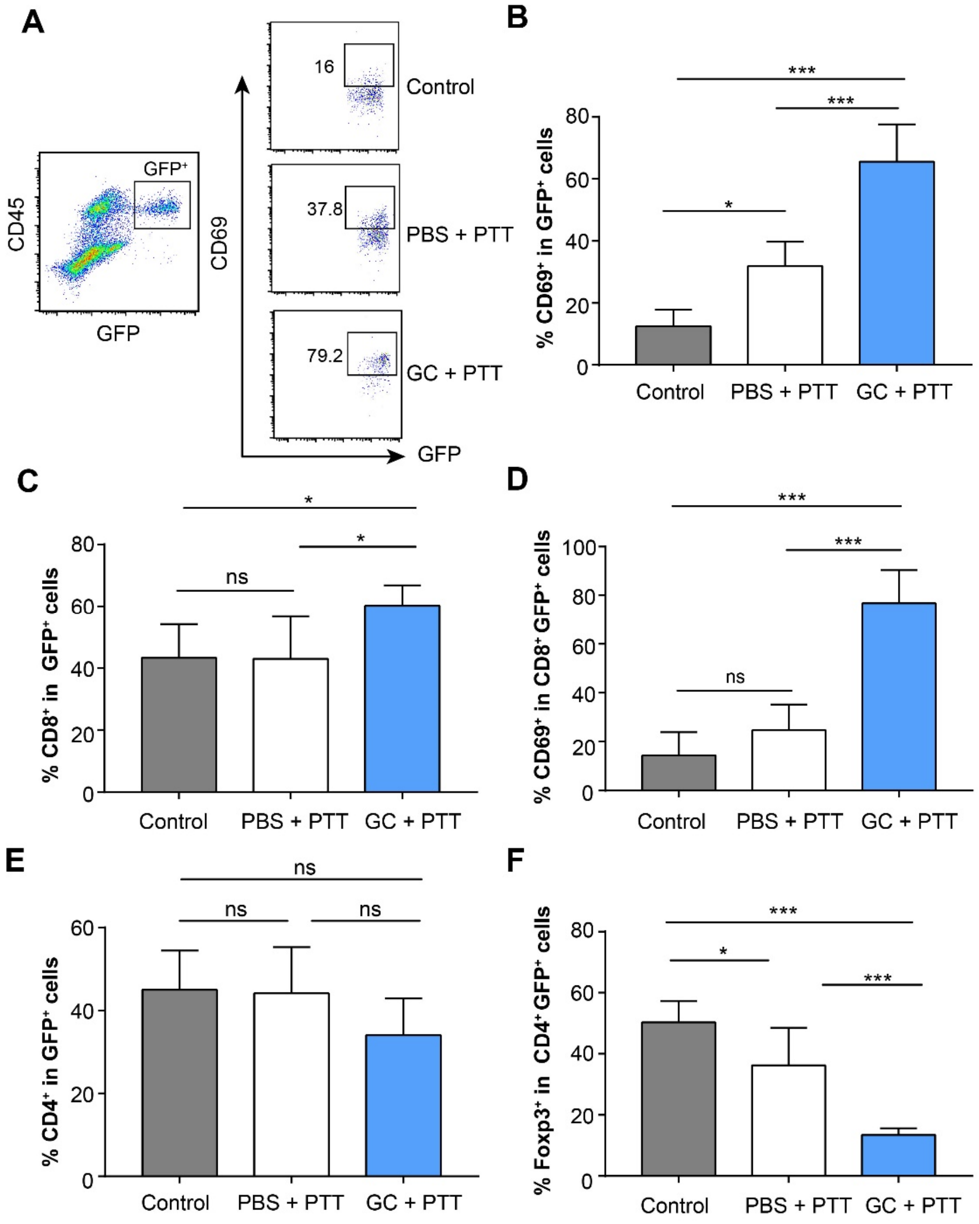

Figure S7. **Characterization of GFP+ TILs in CXCR6-GFP mice re-challenged by CFP-B16 tumor cells (following the same procedures in Fig. 4A). (A)** Representative flow cytometry plots showing CD69+ cells in GFP+ TILs in the re-challenged CFP-B16 tumors of CXCR6-GFP mice. **(B)** Proportion of CD69+ cells in GFP+ TILs from **(A)**. **(C)** Proportion of CD8+ T cells in GFP+ TILs. **(D)** Proportion of CD69+ cells in CD8+ CTLs from **(C)**. **(E)** Proportion of CD4+ T cells in GFP+ TILs. **(F)** Proportion of Foxp3+ cells in CD4+ T cells from **(E)**. Data are presented as mean ± SD (n = 3-5 mice, two independent experiments). Statistical analysis was performed using the one-way ANOVA test followed by the Bonferroni post-test. * $P < 0.05$, ** $P < 0.01$, *** $P < 0.001$, ns: not significant.

Movie S1: ***In vivo*** **sequential imaging of the movement of endogenous GFP$^+$ TILs in the rechallenged tumor microenvironment of different mice groups.** The 3D time-lapse images were acquired as a 30 μm z-stack. Endogenous GFP$^+$ TILs are shown in green (GFP-labeled), and the B16 tumor cells are shown in blue (CFP-labeled). Scale bar: 50 μm.
